# Supplementary material for: Development of an ObLiGaRe Doxycycline Inducible Cas9 system for pre-clinical cancer drug discovery
Source: Nat Commun. 2020 Sep 29;11:4903. doi: 10.1038/s41467-020-18548-9 (PMC7525522; doi:10.1038/s41467-020-18548-9)
Supplement: Supplementary file 1 — Supplementary Information [file 41467_2020_18548_MOESM1_ESM.pdf]

Supplementary Information

Development of an ObLiGaRe doxycycline Inducible Cas9 system for pre-clinical cancer drug discovery

Anders Lundin[1†*], Michelle J. Porritt[1†*], Himjyot Jaiswal[1,8], Frank Seeliger[2], Camilla Johansson[3], Abdel Wahad Bidar[3], Lukas Badertscher[1], Sandra Wimberger[1], Emma J. Davies[4,9], Elizabeth Hardaker[4], Carla P. Martins[4], Emily James[4], Therese Admyre[1], Amir Taheri-Ghahfarokhi[1], Jenna Bradley[5], Anna Schantz[6], Babak Alaeimahabadi[7], Maryam Clausen[1], Xiufeng Xu[1], Lorenz M. Mayr[1], Roberto Nitsch[1], Mohammad Bohlooly-Y[1], Simon T. Barry[4], Marcello Maresca[1*]

† These authors contributed equally to this work

* Corresponding author

1 Translational Genomics, Discovery Sciences, BioPharmaceuticals R&D, AstraZeneca, Gothenburg, Sweden
2 Clinical Pharmacology & Safety Sciences, BioPharmaceuticals R&D, AstraZeneca, Gothenburg, Sweden
3 Clinical Pharmacology & Safety Sciences, Sweden Imaging Hub, BioPharmaceuticals R&D, AstraZeneca, Gothenburg, Sweden
4 Early Oncology TDE, Oncology R&D, AstraZeneca, Li KaShing Centre, Cambridge, UK
5 Discovery Sciences, BioPharmaceuticals R&D, AstraZeneca, Cambridge Science Park, Cambridge, UK
6 Pharmaceutical Sciences, Discovery Sciences, BioPharmaceuticals R&D, AstraZeneca, Gothenburg, Sweden
7 Data Sciences & Quantitative Biology, Discovery Sciences, BioPharmaceuticals R&D, AstraZeneca, Gothenburg, Sweden
8 Current address: Cellink AB, Gothenburg, Sweden
9 Current address: Healx, Cambridge, UK

*Correspondence to:

Marcello Maresca, Translational Genomics, Discovery Sciences, AstraZeneca, Pepparedsleden 1, Mölndal 43 183, Sweden, Phone: +46 31 776 1364

email: Marcello.Maresca@astrazeneca.com

Supplementary Information

Contents

Supplementary Figure 1 3

Supplementary Figure 2 5

Supplementary Figure 3 7

Supplementary Figure 4A 9

Supplementary Figure 4B 11

Supplementary Figure 5 13

Supplementary Note 1 15

Supplementary Note 2 17

Supplementary Table 1 23

Supplementary table 2 24

Supplementary table 3 25

Supplementary table 4 26

Supplementary Information

Supplementary Figure 1

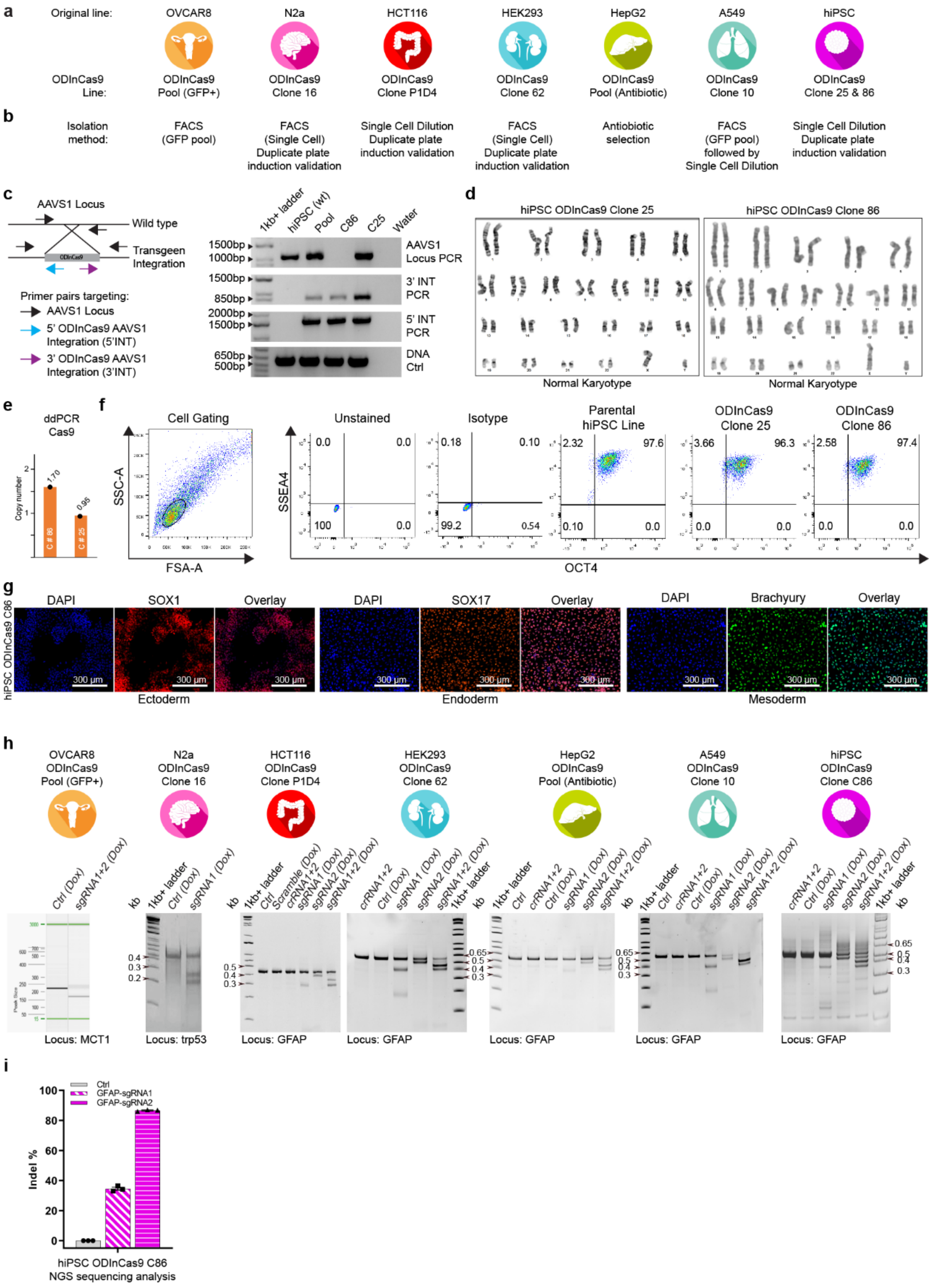

ODInCas9 Manuscript – Supplementary Figure 1 – Lundin A, Porritt M, Maresca M

Supplementary Information

Supplementary Figure 1 (associated to Figure 1) | Cell line generation and validation. (**a**) ODInCas9 cell lines (**b**) isolated by various techniques including FACS of pool GFP+ cells (OVCAR8), single cell FACS with duplicate plate induction validation (N2a, HCT116, HEK293), antibiotic selection (HepG2) and single cell dilution with duplicate plate induction validation (hiPSC). (**c**) AAVS1 locus integrity and AAVS1 ODInCas9 transgene integration of hiPSC, hiPSC ODInCas9 pool, hiPSC ODInCas9 C25 and hiPSC OdinCas9 C86. (**d**) G-banding karyotype evaluation for hiPSC ODInCas9 C25 and C86. (**e**) Copy number integration of ODInCas9 identified by digital droplet PCR probing Cas9 sequence normalized to reference probe AP3B1 (**f**) FACS analysis of pluripotency markers SSEA4 and OCT4 of parental hiPSC line, hiPSC ODInCas9 C25 and C86. (**g**) Differentiation of hiPSC ODInCas9 C86 into the three germ layers assessed by immunocytochemical staining of markers for ectoderm (SOX1), endoderm (SOX17) and mesoderm (TBXT). (**h**) ODInCas9 induction and nuclease activity assessed 48h post transfection by mismatch endonuclease activity assay targeting; MCT1 in OVCAR8 by paired sgRNA, trp53 in N2A by single sgRNA, GFAP in HCT116, HEK293, HepG2, A549 and hiPSC by single sgRNA and paired sgRNAs. (**i**) Nuclease activity assessed 48h post transfection with single sgRNA of ODInCas9 hiPSC quantified by next generation sequencing (n=3 per condition). Dox = Doxycycline induced cell cultures. Scale bar: 300 µm

Supplementary Information

Supplementary Figure 2

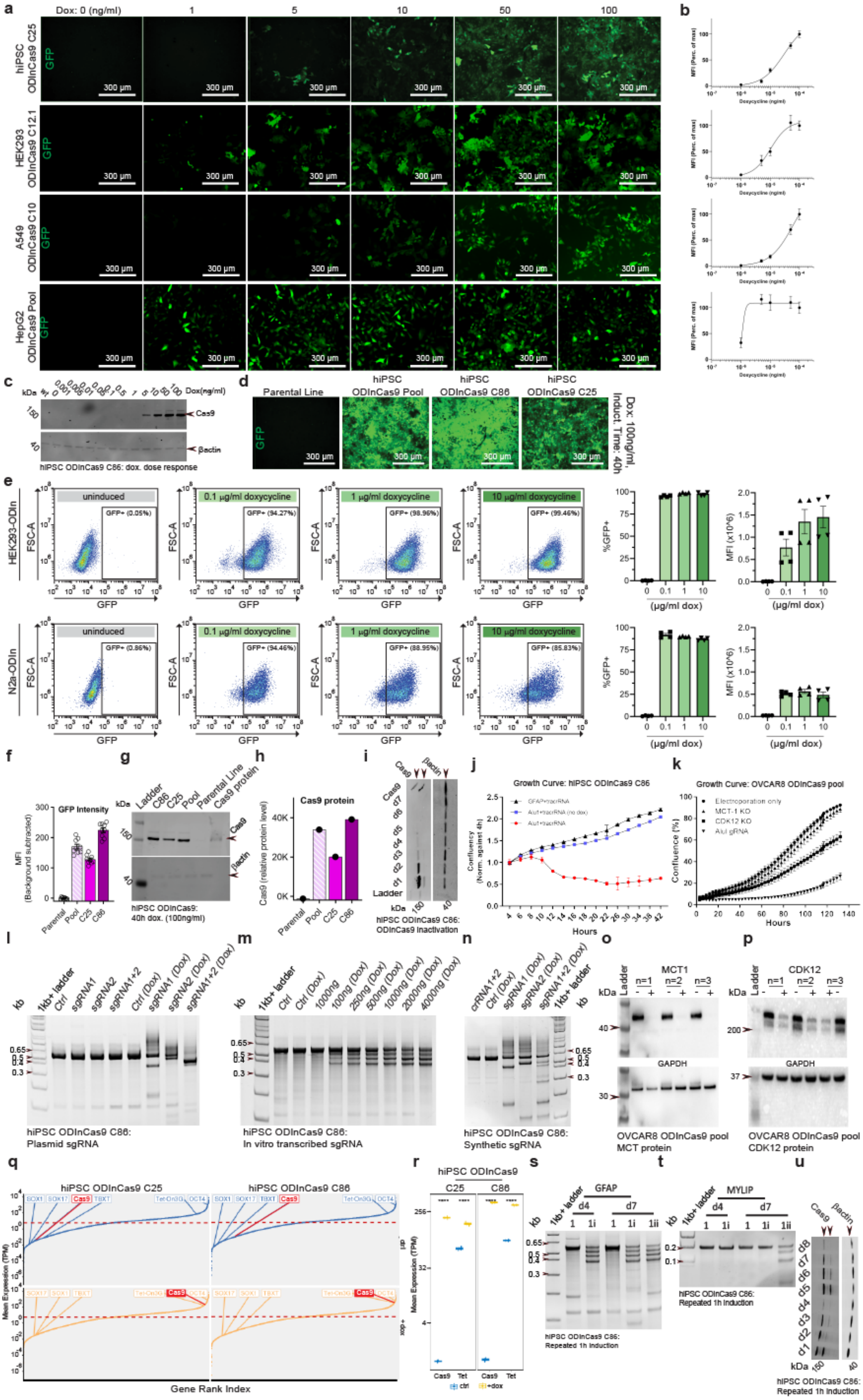

ODInCas9 Manuscript – Supplementary Figure 2 – Lundin A, Porritt M, Maresca M

Supplementary Figure 2 (associated to Figure 2) | Regulation and application of ODInCas9 system. Transgene activation of ODInCas9 lines assessed by (**a**) GFP expression and (**b**) MFI (imaging, n=9 per concentration) normalized to maximum fluorescence intensity at highest dox concentration). (**c**) Cas9 protein expression of ODInCas9.C86 treated with dox concentration 0-100 ng/ml. (**d**) GFP expression of hiPSC parental line, ODInCas9.Pool, ODInCas9.C86/C25 treated with 100ng/ml for 40h. (**e**) Fluorescent analysis of HEK293.C12.1 and N2A.C.16. 48 hr post dox treatment;0.1, 1 and 10 µg/ml (n=4 per concentration, mean ± SEM). (**f**) GFP MFI of hiPSC parental line, ODInCas9.Pool, ODInCas9.C86/C25 treated with 100 ng/ml for 40 hr (imaging, n=9 per concentration; background subtracted, mean ± SEM). (**g**) Cas9 protein expression and (**h**) quantification of hiPSC parental line, ODInCas9.Pool, ODInCas9.C86/C25 treated with 100 ng/ml for 40 hr (normalized to actin concentration). (**i**) Time course study of Cas9 protein expression following 1 hr of 10 µg/ml dox induction. (**j**) Cell confluency normalized against earliest readout of seeding density at 4 hr. Transfection at 0 hr of sgRNA targeting GFAP locus (control) and Alu sites with and w/o dox, 100 ng/ml (n=3 for each condition, each replicate imaged across 15 time points, mean ± SEM). (**k**) Cell confluency following electroporation at 0 hr of sgRNA targeting MCT1, CDK12 and Alu sites (n=3 for each condition, each replicate imaged across 45 time points, mean ± SEM). Mismatch endonuclease activity assay of GFAP locus PCR product targeted by sgRNA and paired sgRNA delivered as (**l**) plasmid sgRNA using liposomal vehicle, (**m**) *in vitro* transcribed sgRNA using lipid nanoparticles at increasing concentrations and (**n**) synthetic sgRNA using liposomal vehicle. (**o**) MCT1 and (**p**) CDK12 protein expression following electroporation at 0 hr of sgRNA targeting MCT1 and CDK12 of activated and non-activated OVCAR8.ODInCas9.Pool cells. (**q**) Expression of *Cas9* and *Tet-On3G* transcript level relative to whole transcriptome in relation to biomarkers *SOX1*, *SOX17*, *TBXT* and *OCT4* of hiPSC.ODInCas9.C25/C86 in the absence or 48 hr treatment with dox (10 µg/ml). Genes without expression in any replicate were filtered out. Only protein coding genes are shown. Mean expression of genes across the 3 replicate is shown on the y axis. (**r**) *Cas9* and *Tet-On3G* transcript levels (TPM) of hiPSC.ODInCas9.C25/C86 in the absence or 48 hr treatment with dox (10 µg/ml). Mismatch endonuclease activity assay of (**s**) GFAP and (**t**) MYLIP locus PCR product targeted by paired sgRNA. Transfection of sgRNAs targeting GFAP and MYLIP performed at d0 and d4, respectively, and genomic DNA isolated at d4 and d7. (**u**) Time course study of Cas9 protein expression during repeated activation of ODInCas9 system by 10 µg/ml dox 1 hr inductions at d0 and d4. Data shown as mean ± SEM, MFI = mean fluorescent intensity, TPM = transcripts per million. **** = <1e-4. Scale bar: 300 µm

Supplementary Figure 3

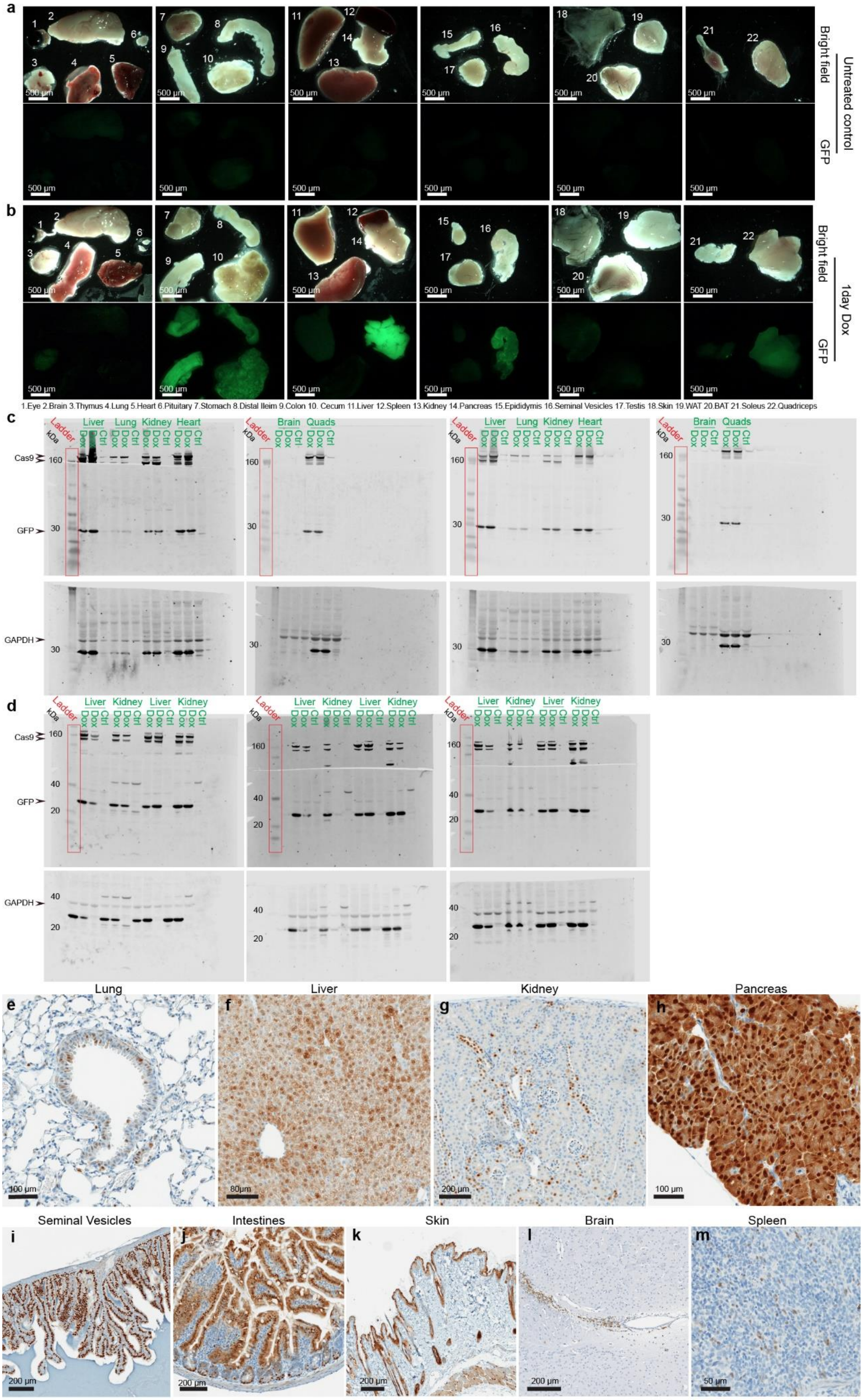

ODInCas9 Manuscript - Supplementary Figure 3 - LundinA. MichelleP. MarescaM.

Supplementary Information

Supplementary Figure 3 (associated to Figure 3) | Regulation of the ODInCas9 system *in vivo*. Evaluation of organ GFP intensity of (**a**) untreated control (**b**) 1 day dox stimulation. (**c**) Western blots of Cas9, GFP and GAPDH for liver, lung, kidney, heart, brain, quadriceps of heterozygote ODInCas9 mice without or with 3-day doxycycline stimulation. (**d**) Western blots of Cas9, GFP and GAPDH for liver and kidney of homozygous, heterozygous ODInCas9 mice and wild type mice after 3-day doxycycline stimulation. Immunohistochemistry section of (**e**) lung, (**f**) liver, (**g**) kidney, (**h**) pancreas, (**i**) seminal vesicles, (**j**) intestines, (**k**) skin, (**l**) brain and (**m**) spleen staining for Cas9 in heterozygote ODInCas9 mice.

Supplementary Information

Supplementary Figure 4A

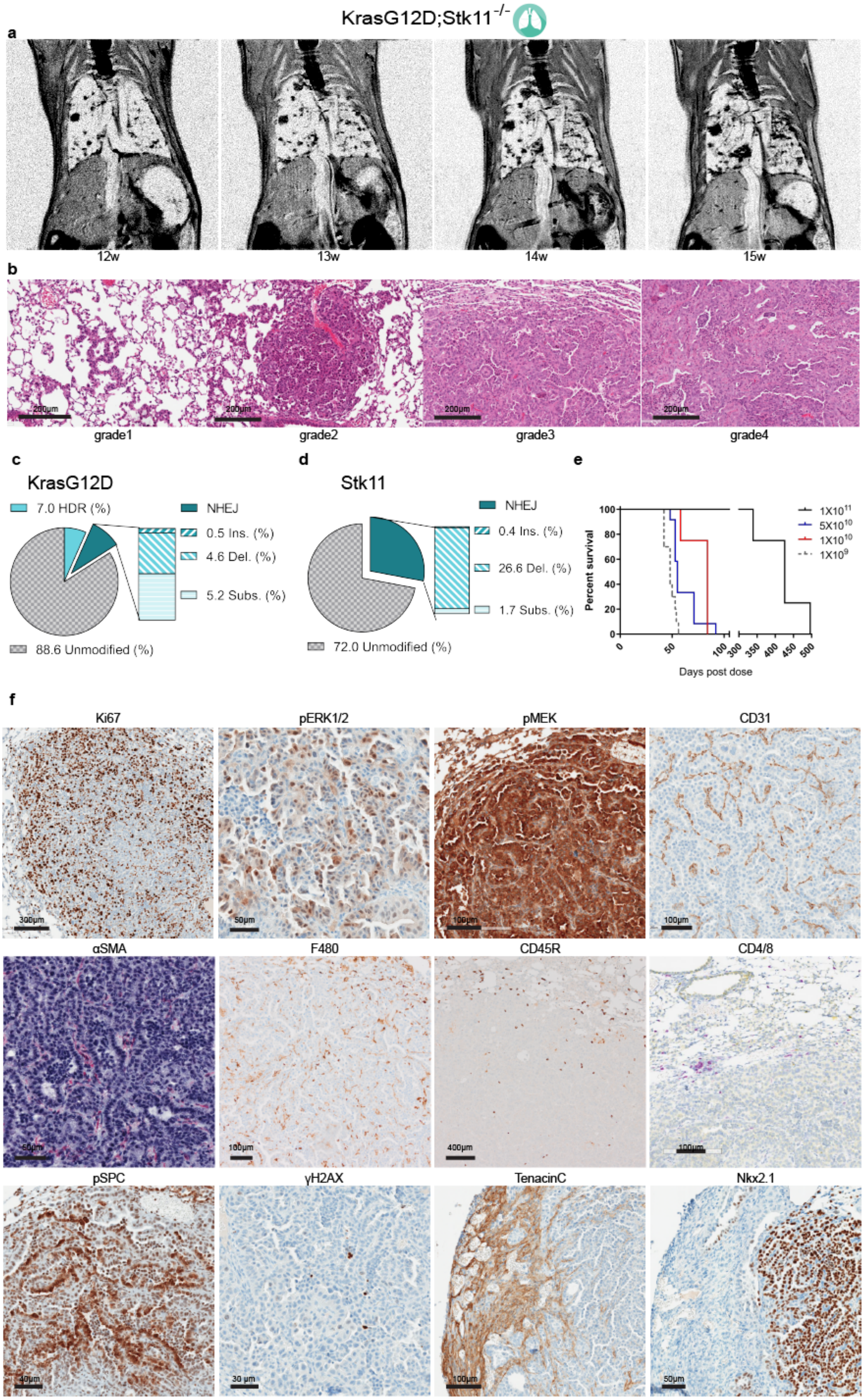

ODInCas9 Manuscript – Supplementary Figure 4A – Lundin A, Porritt M, Maresca M

Supplementary Information

Supplementary Figure 4A (associated to Figure 4) | Modelling NSCLC in the ODInCas9 *KrasG12D;Stk11$^{-/-}$* model. (**a**) Time course of tumor development by MRI following the same mice (**b**) Representative HE staining of mouse lung capturing the various stages of lung adenocarcinoma (**c,d**) Classification of amplicon sequencing of lung cancer tissue at *Kras* and *Stk11* locus (**e**) Kaplan-Meier survival curve for 4 different AAV9 viral titers (GC) (**f**) Immunohistochemistry section of lung tumors staining for Ki67, pERK, pMEK, CD31, αSMA, F480, CD45R, CD4/8, pSPC, ɣH2AX, Tenascin C and Nkx2.1.

Supplementary Information

Supplementary Figure 4B

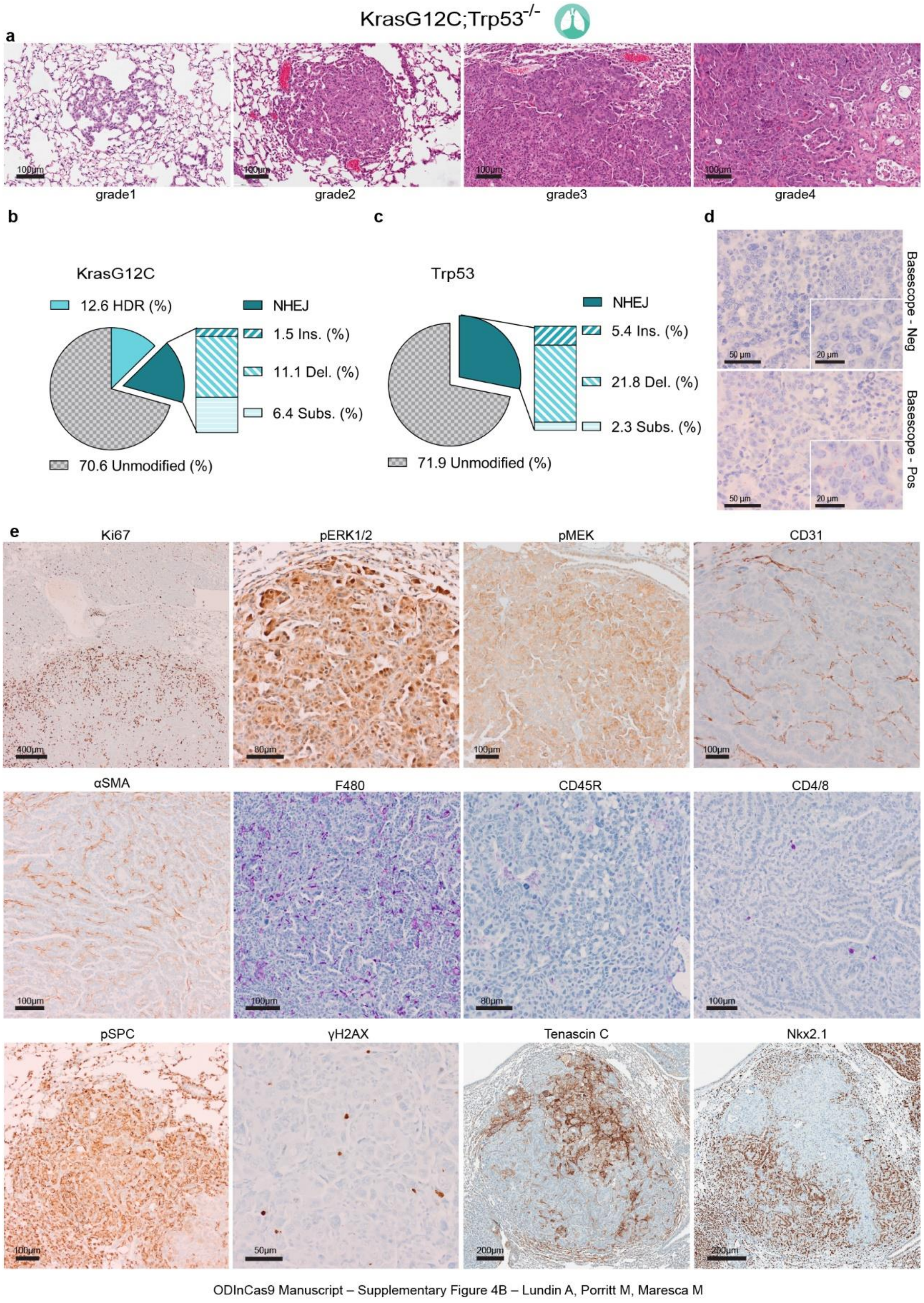

ODInCas9 Manuscript – Supplementary Figure 4B – Lundin A, Porritt M, Maresca M

Supplementary Information

Supplementary Figure 4B (associated to Figure 4) ODInCas9 *KrasG12C;Trp53*$^{-/-}$ NSCLC model. (**a**) Representative HE staining of mouse lung capturing the various stages of lung adenocarcinoma (**b,c**) Classification of amplicon sequencing of lung cancer tissue at *Kras* and *Trp53* locus (**d**) DNA *in situ* hybridization targeting KrasG12C (**e**) Immunohistochemistry staining of lung tumors for Ki67, pERK, pMEK, CD31, αSMA, F480, CD45R, CD4/8, pSPC, ɣH2AX, Tenascin C and Nkx2.1

Supplementary Figure 5

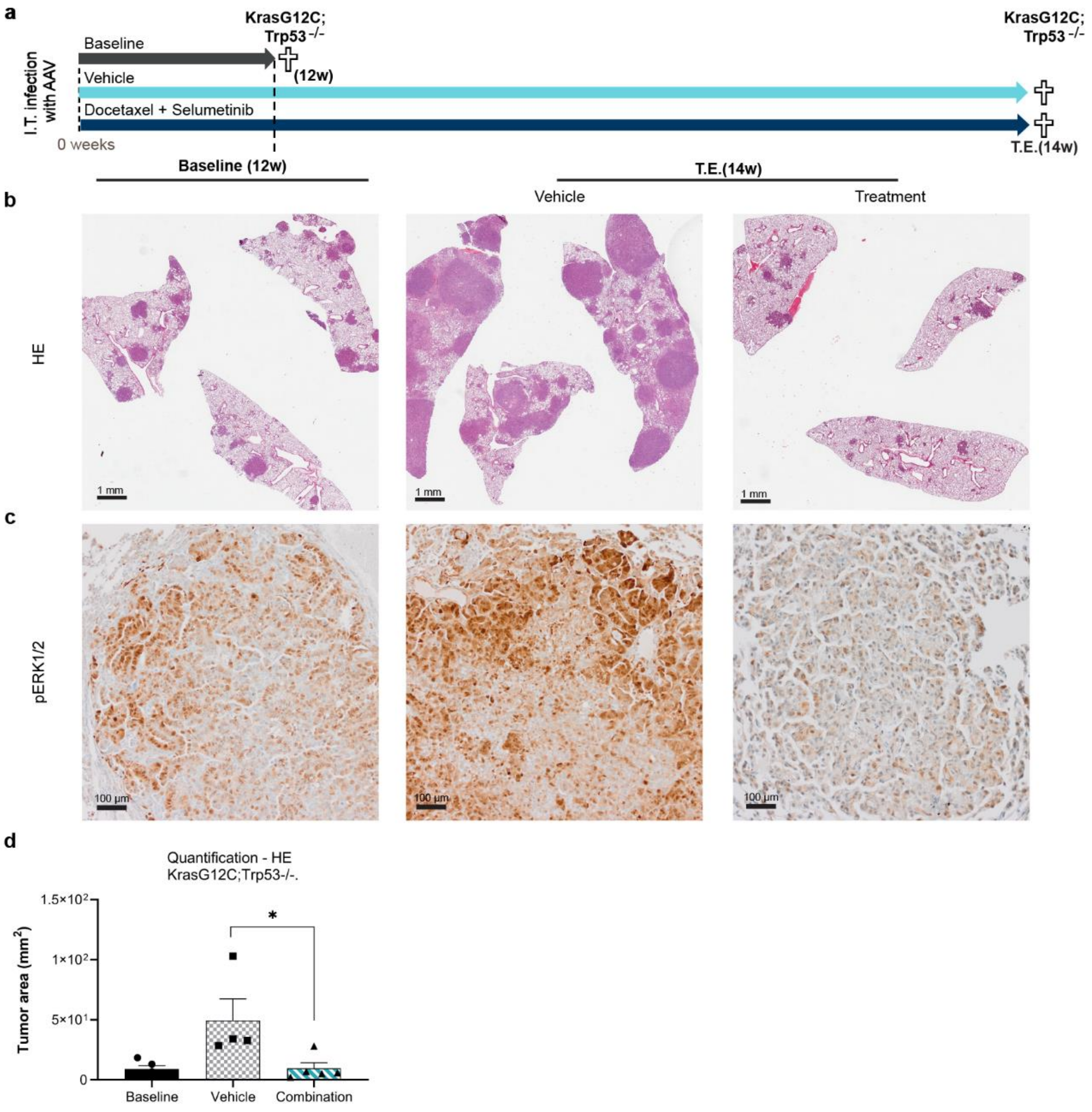

ODInCas9 Manuscript – Supplementary Figure 5A – Lundin A, Porritt M, Maresca M

Supplementary Information

Supplementary Figure 5 (associated to Figure 5) | Pre-clinical efficacy study of ODInCas9 *KrasG12C;Trp53*$^{-/-}$ NSCLC model (a) Timeline for treatment study. ODInCas9 mice (n=22) were induced with dox, dosed with AAV and allocated to treatment arm at 12 weeks when significant tumor burden is observed. Mice were treated for 2-weeks with either vehicle or Docetaxel + Selumetinib combination treatment. Evaluation at baseline and treatment end (T.E) by (**b**) lung tissue histology (HE) (**c**) pERK1/2 immunohistochemical staining. (**d**) Quantification of tumor burden at baseline, vehicle and combinatory treatment using histology (one-way ANOVA, $F(2,11) = 5.756$, $p=0.0195$). Experimental set-up included baseline (n=5), vehicle (n=4) and combination (n=5). Data are presented as mean values +/-SEM.

Supplementary Information

Supplementary Note 1

AAVS1 pZFN1-2A-ZFN2 (highlighted Left ZFN-2A-Right ZFN)

GACTCTTCGCGATGTACGGGCCAGATATACGCGTTGACATTGATTATTGACTAGTTATTAATAGTAATCAATTACGGGGTC
ATTAGTTCATAGCCCATATATGGAGTTCCGCGTTACATAACTTACGGTAAATGGCCCGCCTGGCTGACCGCCCAACGACCC
CCGCCCATTGACGTCAATAATGACGTATGTTCCCATAGTAACGCCAATAGGGACTTTCCATTGACGTCAATGGGTGGACTA
TTTACGGTAAACTGCCCACTTGGCAGTACATCAAGTGTATCATATGCCAAGTACGCCCCCTATTGACGTCAATGACGGTAA
ATGGCCCGCCTGGCATTATGCCCAGTACATGACCTTATGGGACTTTCCTACTTGGCAGTACATCTACGTATTAGTCATCGCT
ATTACCATGGTGATGCGGTTTTGGCAGTACATCAATGGGCGTGGATAGCGGTTTGACTCACGGGGATTTCCAAGTCTCCAC
CCCATTGACGTCAATGGGAGTTTGTTTTGGCACCAAAATCAACGGGACTTTCCAAAATGTCGTAACAACTCCGCCCCATTG
ACGCAAATGGGCGGTAGGCGTGTACGGTGGGAGGTCTATATAAGCAGAGCTCTCTGGCTAACTAGAGAACCCACTGCTTA
CTGGCTTATCGAAATTAATACGACTCACTATAGGGAGACCCAAGCTGGCTAGCGTTTAAACTTAAGCTGATCCACTAGTCC
AGTGTGGTGGAATTCGCCATGGACTACAAAGACCATGACGGTGATTATAAAGATCATGACATCGATTACAAGGATGACGA
TGACAAGATGGCCCCCAAGAAGAAGAGGAAGGTGGGCATCCACGGGGTACCCGCCGCTATGGCTGAGAGGCCCTTCCAG
TGTCGAATCTGCATGCGTAACTTCAGTTACAACTGGCACCTGCAGCGCCACATCCGCACCCACACAGGCGAGAAGCCTTTT
GCCTGTGACATTTGTGGGAGGAAGTTTGCCCGCTCCGACCACCTGACCACCCATACCAAGATACACACGGGATCTCAGAA
GCCCTTCCAGTGTCGAATCTGCATGCGTAACTTCAGTCACAACTACGCCCGCGACTGTCACATCCGCACCCACACAGGCGA
GAAGCCTTTTGCCTGTGACATTTGTGGGAGGAAGTTTGCCCAGAACTCCACCCGCATCGGCCATACCAAGATACACCTGCG
GGGATCCCAGCTGGTGAAGAGCGAGCTGGAGGAGAAGAAGTCCGAGCTGCGGCACAAGCTGAAGTACGTGCCCCACGA
GTACATCGAGCTGATCGAGATCGCCAGGAACAGCACCCAGGACCGCATCCTGGAGATGAAGGTGATGGAGTTCTTCATGA
AGGTGTACGGCTACAGGGGAAAGCACCTGGGCGGAAGCAGAAAGCCTGACGGCGCCATCTATACAGTGGGCAGCCCCAT
CGATTACGGCGTGATCGTGGACACAAAGGCCTACAGCGGCGGCTACAATCTGCCTATCGGCCAGGCCGACGAGATGGAG
AGATACGTGGAGGAGAACCAGACCCGGAATAAGCACCTCAACCCCAACGAGTGGTGGAAGGTGTACCCTAGCAGCGTGA
CCGAGTTCAAGTTCCTGTTCGTGAGCGGCCACTTCAAGGGCAACTACAAGGCCCAGCTGACCAGGCTGAACCACATCACCA
ACTGCAATGGCGCCGTGCTGAGCGTGGAGGAGCTGCTGATCGGCGGCGAGATGATCAAAGCCGGCACCCTGACACTGGA
GGAGGTGCGGCGCAAGTTCAACAACGGCGAGATCAACTTCAGATCTGAGGGCAGAGGAAGTCTGCTAACATGCGGTGAC
GTCGAGGAGAATCCTGGCCCAGACTACAAAGACCATGACGGTGATTATAAAGATCATGACATCGATTACAAGGATGACGA
TGACAAGATGGCCCCCAAGAAGAAGAGGAAGGTGGGCATTCATGGGGTACCCGCCGCTATGGCTGAGAGGCCCTTCCAG
TGTCGAATCTGCATGCGTAACTTCAGTCAGTCCTCCAACCTGGCCCGCCACATCCGCACCCACACAGGCGAGAAGCCTTTT
GCCTGTGACATTTGTGGGAGGAAGTTTGCCCGCACCGACTACCTGGTGGACCATACCAAGATACACACGGGATCTCAGAA
GCCCTTCCAGTGTCGAATCTGCATGCGTAACTTCAGTTACAACACCCACCTGACCCGCCACATCCGCACCCACACAGGCGA
GAAGCCTTTTGCCTGTGACATTTGTGGGAGGAAGTTTGCCCAGGGCTACAACCTGGCCGGCCATACCAAGATACACCTGC
GGGGATCCCAGCTGGTGAAGAGCGAGCTGGAGGAGAAGAAGTCCGAGCTGCGGCACAAGCTGAAGTACGTGCCCCACG
AGTACATCGAGCTGATCGAGATCGCCAGGAACAGCACCCAGGACCGCATCCTGGAGATGAAGGTGATGGAGTTCTTCATG
AAGGTGTACGGCTACAGGGGAAAGCACCTGGGCGGAAGCAGAAAGCCTGACGGCGCCATCTATACAGTGGGCAGCCCCA
TCGATTACGGCGTGATCGTGGACACAAAGGCCTACAGCGGCGGCTACAATCTGCCTATCGGCCAGGCCGACGAGATGCA
GAGATACGTGAAGGAGAACCAGACCCGGAATAAGCACATCAACCCCAACGAGTGGTGGAAGGTGTACCCTAGCAGCGTG
ACCGAGTTCAAGTTCCTGTTCGTGAGCGGCCACTTCAAGGGCAACTACAAGGCCCAGCTGACCAGGCTGAACCACAAAAC
CAACTGCAATGGCGCCGTGCTGAGCGTGGAGGAGCTGCTGATCGGCGGCGAGATGATCAAAGCCGGCACCCTGACACTG
GAGGAGGTGCGGCGCAAGTTCAACAACGGCGAGATCAACTTCAGATCTTGATAACTCGAGTCTAGAGGGCCCGTTTAAAC
CCGCTGATCAGCCTCGACTGTGCCTTCTAGTTGCCAGCCATCTGTTGTTTGCCCCTCCCCCGTGCCTTCCTTGACCCTGGAAG
GTGCCACTCCCACTGTCCTTTCCTAATAAAATGAGGAAATTGCATCGCATTGTCTGAGTAGGTGTCATTCTATTCTGGGGGG
TGGGGTGGGGCAGGACAGCAAGGGGGAGGATTGGGAAGACAATAGCAGGCATGCTGGGGATGCGGTGGGCTCTATGG
CTTCTACTGGGCGGTTTTATGGACAGCAAGCGAACCGGAATTGCCAGCTGGGGCGCCCTCTGGTAAGGTTGGGAAGCCCT
GCAAAGTAAACTGGATGGCTTTCTCGCCGCCAAGGATCTGATGGCGCAGGGGATCAAGCTCTGATCAAGAGACAGGATG
AGGATCGTTTCGCATGATTGAACAAGATGGATTGCACGCAGGTTCTCCGGCCGCTTGGGTGGAGAGGCTATTCGGCTATG
ACTGGGCACAACAGACAATCGGCTGCTCTGATGCCGCCGTGTTCCGGCTGTCAGCGCAGGGGCGCCCGGTTCTTTTTGTCA
AGACCGACCTGTCCGGTGCCCTGAATGAACTGCAAGACGAGGCAGCGCGGCTATCGTGGCTGGCCACGACGGGCGTTCC
TTGCGCAGCTGTGCTCGACGTTGTCACTGAAGCGGGAAGGGACTGGCTGCTATTGGGCGAAGTGCCGGGGCAGGATCTC
CTGTCATCTCACCTTGCTCCTGCCGAGAAAGTATCCATCATGGCTGATGCAATGCGGCGGCTGCATACGCTTGATCCGGCT
ACCTGCCCATTCGACCACCAAGCGAAACATCGCATCGAGCGAGCACGTACTCGGATGGAAGCCGGTCTTGTCGATCAGGA

TGATCTGGACGAAGAGCATCAGGGGCTCGCGCCAGCCGAACTGTTCGCCAGGCTCAAGGCGAGCATGCCCGACGGCGAG
GATCTCGTCGTGACCCATGGCGATGCCTGCTTGCCGAATATCATGGTGGAAAATGGCCGCTTTTCTGGATTCATCGACTGT
GGCCGGCTGGGTGTGGCGGACCGCTATCAGGACATAGCGTTGGCTACCCGTGATATTGCTGAAGAGCTTGGCGGCGAAT
GGGCTGACCGCTTCCTCGTGCTTTACGGTATCGCCGCTCCCGATTCGCAGCGCATCGCCTTCTATCGCCTTCTTGACGAGTT
CTTCTGAATTATTAACGCTTACAATTTCCTGATGCGGTATTTTCTCCTTACGCATCTGTGCGGTATTTCACACCGCATACAGG
TGGCACTTTTCGGGGAAATGTGCGCGGAACCCCTATTTGTTTATTTTTCTAAATACATTCAAATATGTATCCGCTCATGAGA
CAATAACCCTGATAAATGCTTCAATAATAGCACGTGCTAAAACTTCATTTTTAATTTAAAAGGATCTAGGTGAAGATCCTTT
TTGATAATCTCATGACCAAAATCCCTTAACGTGAGTTTTCGTTCCACTGAGCGTCAGACCCCGTAGAAAAGATCAAAGGAT
CTTCTTGAGATCCTTTTTTTCTGCGCGTAATCTGCTGCTTGCAAACAAAAAAACCACCGCTACCAGCGGTGGTTTGTTTGCC
GGATCAAGAGCTACCAACTCTTTTTCCGAAGGTAACTGGCTTCAGCAGAGCGCAGATACCAAATACTGTCCTTCTAGTGTA
GCCGTAGTTAGGCCACCACTTCAAGAACTCTGTAGCACCGCCTACATACCTCGCTCTGCTAATCCTGTTACCAGTGGCTGCT
GCCAGTGGCGATAAGTCGTGTCTTACCGGGTTGGACTCAAGACGATAGTTACCGGATAAGGCGCAGCGGTCGGGCTGAA
CGGGGGGTTCGTGCACACAGCCCAGCTTGGAGCGAACGACCTACACCGAACTGAGATACCTACAGCGTGAGCTATGAGA
AAGCGCCACGCTTCCCGAAGGGAGAAAGGCGGACAGGTATCCGGTAAGCGGCAGGGTCGGAACAGGAGAGCGCACGAG
GGAGCTTCCAGGGGGAAACGCCTGGTATCTTTATAGTCCTGTCGGGTTTCGCCACCTCTGACTTGAGCGTCGATTTTTGTG
ATGCTCGTCAGGGGGGCGGAGCCTATGGAAAAACGCCAGCAACGCGGCCTTTTTACGGTTCCTGGGCTTTTGCTGGCCTTT
TGCTCACATGTTCTT

Supplementary Note 2

ODIn-inv-neo-Cas9 for mouse model (highlighted AAVS1oblZFN site Rosa26oblZFN site Insulator Cas9)

GTGGCACTTTTCGGGGAAATGTGCGCGGAACCCCTATTTGTTTATTTTTCTAAATACATTCAAATA
TGTATCCGCTCATGAGACAATAACCCTGATAAATGCTTCAATAATATTGAAAAAGGAAGAGTATGA
GTATTCAACATTTCCGTGTCGCCCTTATTCCCTTTTTTGCGGCATTTTGCCTTCCTGTTTTTGCTCA
CCCAGAAACGCTGGTGAAAGTAAAAGATGCTGAAGATCAGTTGGGTGCACGAGTGGGTTACATC
GAACTGGATCTCAACAGCGGTAAGATCCTTGAGAGTTTTCGCCCCGAAGAACGTTTTCCAATGAT
GAGCACTTTTAAAGTTCTGCTATGTGGCGCGGTATTATCCCGTATTGACGCCGGGCAAGAGCAAC
TCGGTCGCCGCATACACTATTCTCAGAATGACTTGGTTGAGTACTCACCAGTCACAGAAAAGCAT
CTTACGGATGGCATGACAGTAAGAGAATTATGCAGTGCTGCCATAACCATGAGTGATAACACTGC
GGCCAACTTACTTCTGACAACGATCGGAGGACCGAAGGAGCTAACCGCTTTTTTGCACAACATGG
GGGATCATGTAACTCGCCTTGATCGTTGGGAACCGGAGCTGAATGAAGCCATACCAAACGACGA
GCGTGACACCACGATGCCTGTAGCAATGGCAACAACGTTGCGCAAACTATTAACTGGCGAACTAC
TTACTCTAGCTTCCCGGCAACAATTAATAGACTGGATGGAGGCGGATAAAGTTGCAGGACCACTT
CTGCGCTCGGCCCTTCCGGCTGGCTGGTTTATTGCTGATAAATCTGGAGCCGGTGAGCGTGGGT
CTCGCGGTATCATTGCAGCACTGGGGCCAGATGGTAAGCCCTCCCGTATCGTAGTTATCTACAC
GACGGGGAGTCAGGCAACTATGGATGAACGAAATAGACAGATCGCTGAGATAGGTGCCTCACTG
ATTAAGCATTGGTAACTGTCAGACCAAGTTTACTCATATATACTTTAGATTGATTTAAAACTTCATTT
TTAATTTAAAAGGATCTAGGTGAAGATCCTTTTTGATAATCTCATGACCAAAATCCCTTAACGTGAG
TTTTCGTTCCACTGAGCGTCAGACCCCGTAGAAAAGATCAAAGGATCTTCTTGAGATCCTTTTTTT
CTGCGCGTAATCTGCTGCTTGCAAACAAAAAAACCACCGCTACCAGCGGTGGTTTGTTTGCCGGA
TCAAGAGCTACCAACTCTTTTTCCGAAGGTAACTGGCTTCAGCAGAGCGCAGATACCAAATACTG
TTCTTCTAGTGTAGCCGTAGTTAGGCCACCACTTCAAGAACTCTGTAGCACCGCCTACATACCTC
GCTCTGCTAATCCTGTTACCAGTGGCTGCTGCCAGTGGCGATAAGTCGTGTCTTACCGGGTTGG
ACTCAAGACGATAGTTACCGGATAAGGCGCAGCGGTCGGGCTGAACGGGGGGTTCGTGCACAC
AGCCCAGCTTGGAGCGAACGACCTACACCGAACTGAGATACCTACAGCGTGAGCTATGAGAAAG
CGCCACGCTTCCCGAAGGGAGAAAGGCGGACAGGTATCCGGTAAGCGGCAGGGTCGGAACAGG
AGAGCGCACGAGGGAGCTTCCAGGGGGAAACGCCTGGTATCTTTATAGTCCTGTCGGGTTTCGC
CACCTCTGACTTGAGCGTCGATTTTTGTGATGCTCGTCAGGGGGGCGGAGCCTATGGAAAAACG
CCAGCAACGCGGCCTTTTTACGGTTCCTGGCCTTTTGCTGGCCTTTTGCTCACATGTTCTTTCCTG
CGTTATCCCCTGATTCTGTGGATAACCGTATTACCGCCTTTGAGTGAGCTGATACCGCTCGCCGC
AGCCGAACGACCGAGCGCAGCGAGTCAGTGAGCGAGGAAGCGGAAGAGCGCCCAATACGCAAA
CCGCCTCTCCCCGCGCGTTGGCCGATTCATTAATGCAGCTGGCACGACAGGTTTCCCGACTGGA
AAGCGGGCAGTGAGCGCAACGCAATTAATGTGAGTTAGCTCACTCATTAGGCACCCCAGGCTTTA
CACTTTATGCTTCCGGCTCGTATGTTGTGTGGAATTGTGAGCGGATAACAATTTCACACAGGAAA
CAGCTATGACCATGATTACGCCAAGCGCGCAATTAACCCTCACTAAAGGGAACCTCCCCTAGCTT
AATTAACCCTAGAAAGATAATCATATTGTGACGTACGTTAAAGATAATCATGCGTAAAATTGACGC
ATGTGTTTTATCGATCTGTATATCGAGGTTTATTTATTAATTTGAATAGATATTAAGTTTTATTATATT
TACACTTACATACTAATAATAAATTCAACAAACAATTTATTTATGTTTATTTATTTATTAAAAAAAAAC
AAAAACTCAAAATTTCTTCTATAAAGTAACAAAACTTTTAAACATTCTCTCTTTTACAAAAATAAACT
TATTTTGTACTTTAAAAACAGTCATGTTGTATTATAAAATAAGTAATTAGCTTAACTTATACATAATA
GAAACAAATTATACTTATTAATCGCATTGATTATTGACTAGTCGTATTAAGGGTTCCGGATCAGCTT
GATTCGAGCCCCAGCTGGTTCTTTCCGCCTCAGAAGCCATAGAGCCCACCGCATCCCCAGCATG
CCTGCTATTGTCTTCCCAATCCTCCCCCTTGCTGTCCTGCCCCACCCCACCCCCCAGAATAGAAT
GACACCTACTCAGACAATGCGATGCAATTTCCTCATTTTATTAGGAAAGGACAGTGGGAGTGGCA
CCTTCCAGGGTCAAGGAAGGCACGGGGGAGGGGCAAACAACAGATGGCTGGCAACTAGAAGGC
ACAGTCGAGGCTGATCAGCGAGCTCTAGAGAATTGATCCCCTCAGAAGAACTCGTCAAGAAGGC
GATAGAAGGCGATGCGCTGCGAATCGGGAGCGGCGATACCGTAAAGCACGAGGAAGCGGTCAG
CCCATTCGCCGCCAAGCTCTTCAGCAATATCACGGGTAGCCAACGCTATGTCCTGATAGCGGTC
CGCCACACCCAGCCGGCCACAGTCGATGAATCCAGAAAAGCGGCCATTTTCCACCATGATATTC
GGCAAGCAGGCATCGCCATGGGTCACGACGAGATCCTCGCCGTCGGGCATGCGCGCCTTGAGC
CTGGCGAACAGTTCGGCTGGCGCGAGCCCCTGATGCTCTTCGTCCAGATCATCCTGATCGACAA

GACCGGCTTCCATCCGAGTACGTGCTCGCTCGATGCGATGTTTCGCTTGGTGGTCGAATGGGCA
GGTAGCCGGATCAAGCGTATGCAGCCGCCGCATTGCATCAGCCATGATGGATACTTTCTCGGCA
GGAGCAAGGTGAGATGACAGGAGATCCTGCCCCGGCACTTCGCCCAATAGCAGCCAGTCCCTTC
CCGCTTCAGTGACAACGTCGAGCACAGCTGCGCAAGGAACGCCCGTCGTGGCCAGCCACGATA
GCCGCGCTGCCTCGTCCTGCAGTTCATTCAGGGCACCGGACAGGTCGGTCTTGACAAAAAGAAC
CGGGCGCCCCTGCGCTGACAGCCGGAACACGGCGGCATCAGAGCAGCCGATTGTCTGTTGTGC
CCAGTCATAGCCGAATAGCCTCTCCACCCAAGCGGCCGGAGAACCTGCGTGCAATCCATCTTGT
TCAATGGCCGATCCCATGGTTTAGTTCCTCACCTTGTCGTATTATACTATGCCGATATACTATGCC
GATGATTAATTGTCAACACGTGCTGCTGCAGGTCGAAAGGCCCGGAGATGAGGAAGAGGAGAAC
AGCGCGGCAGACGTGCGCTTTTGAAGCGTGCAGAATGCCGGGCCTCCGGAGGACCTTCGGGCG
CCCGCCCCGCCCCTGAGCCCGCCCCTGAGCCCGCCCCCGGACCCACCCCTTCCCAGCCTCTGA
GCCCAGAAAGCGAAGGAGCAAAGCTGCTATTGGCCGCTGCCCCAAAGGCCTACCCGCTTCCATT
GCTCAGCGGTGCTGTCCATCTGCACGAGACTAGTGAGACGTGCTACTTCCATTTGTCACGTCCTG
CACGACGCGAGCTGCGGGGCGGGGGGGAACTTCCTGACTAGGGGAGGAGTAGAAGGTGGCGC
GAAGGGGCCACCAAAGAACGGAGCCGGTTGGCGCCTACCGGTGGATGTGGAATGTGTGCGAGC
CAGAGGCCACTTGTGTAGCGCCAAGTGCCCAGCGGGGCTGCTAAAGCGCATGCTCCAGACTGC
CTTGGGAAAAGCGCCTCCCCTACCCGGTAGACACCCCACAGTGGGTGGCCTAGGGACAGGATT
GCAACTCCAGTCTTTCTTCTTCTTGGGCGGGAGTCACTAGTTATTAATAGTAATCAATTACGGGGT
CATTAGTTCATAGCCCATATATGGAGTTCCGCGTTACATAACTTACGGTAAATGGCCCGCCTGGC
TGACCGCCCAACGACCCCCGCCCATTGACGTCAATAATGACGTATGTTCCCATAGTAACGCCAAT
AGGGACTTTCCATTGACGTCAATGGGTGGACTATTTACGGTAAACTGCCCACTTGGCAGTACATC
AAGTGTATCATATGCCAAGTACGCCCCCTATTGACGTCAATGACGGTAAATGGCCCGCCTGGCAT
TATGCCCAGTACATGACCTTATGGGACTTTCCTACTTGGCAGTACATCTACGTATTAGTCATCGCT
ATTACCATGGGTCGAGGTGAGCCCCACGTTCTGCTTCACTCTCCCCATCTCCCCCCCCTCCCCAC
CCCCAATTTTGTATTTATTTATTTTTTAATTATTTTGTGCAGCGATGGGGGCGGGGGGGGGGGGG
GCGCGCGCCAGGCGGGGCGGGGCGGGGCGAGGGGCGGGGCGGGGCGAGGCGGAGAGGTGC
GGCGGCAGCCAATCAGAGCGGCGCGCTCCGAAAGTTTCCTTTTATGGCGAGGCGGCGGCGGCG
GCGGCCCTATAAAAAGCGAAGCGCGCGGCGGGCGGGAGTCGCTGCGTTGCCTTCGCCCCGTGC
CCCGCTCCGCGCCGCCTCGCGCCGCCCGCCCCGGCTCTGACTGACCGCGTTACTCCCACAGGT
GAGCGGGCGGGACGGCCCTTCTCCTCCGGGCTGTAATTAGCGCTTGGTTTAATGACGGCTCGTT
TCTTTTCTGTGGCTGCGTGAAAGCCTTAAAGGGCTCCGGGAGGGCCCTTTGTGCGGGGGGGAG
CGGCTCGGGGGGTGCGTGCGTGTGTGTGTGCGTGGGGAGCGCCGCGTGCGGCCCGCGCTGCC
CGGCGGCTGTGAGCGCTGCGGGCGCGGCGCGGGGCTTTGTGCGCTCCGCGTGTGCGCGAGGG
GAGCGCGGCCGGGGGCGGTGCCCCGCGGTGCGGGGGGGCTGCGAGGGGAACAAAGGCTGCG
TGCGGGGTGTGTGCGTGGGGGGGTGAGCAGGGGGTGTGGGCGCGGCGGTCGGGCTGTAACCC
CCCCCTGCACCCCCCTCCCCGAGTTGCTGAGCACGGCCCGGCTTCGGGTGCGGGGCTCCGTGC
GGGGCGTGGCGCGGGGCTCGCCGTGCCGGGCGGGGGGTGGCGGCAGGTGGGGGTGCCGGG
CGGGGCGGGGCCGCCTCGGGCCGGGGAGGGCTCGGGGGAGGGGCGCGGCGGCCCCGGAGC
GCCGGCGGCTGTCGAGGCGCGGCGAGCCGCAGCCATTGCCTTTTATGGTAATCGTGCGAGAGG
GCGCAGGGACTTCCTTTGTCCCAAATCTGGCGGAGCCGAAATCTGGGAGGCGCCGCCGCACCC
CCTCTAGCGGGCGCGGGCGAAGCGGTGCGGCGCCGGCAGGAAGGAAATGGGCGGGGAGGGC
CTTCGTGCGTCGCCGCGCCGCCGTCCCCTTCTCCATCTCCAGCCTCGGGGCTGCCGCAGGGGG
ACGGCTGCCTTCGGGGGGGACGGGGCAGGGCGGGGTTCGGCTTCTGGCGTGTGACCGGCGGC
TCTAGAGCCTCTGCTAACCATGTTCATGCCTTCTTCTTTTTCCTACAGCTCCTGGGCAACGTGCTG
GTTATTGTGCTGTCTCATCATTTTGGCAAAGAATTCGCCACCATGGTGCCCAAGAAGAAGAGGAA
AGTCTCTAGACTGGACAAGAGCAAAGTCATAAACTCTGCTCTGGAATTACTCAATGGAGTCGGTA
TCGAAGGCCTGACGACAAGGAAACTCGCTCAAAAGCTGGGAGTTGAGCAGCCTACCCTGTACTG
GCACGTGAAGAACAAGCGGGCCCTGCTCGATGCCCTGCCAATCGAGATGCTGGACAGGCATCAT
ACCCACTCCTGCCCCCTGGAAGGCGAGTCATGGCAAGACTTTCTGCGGAACAACGCCAAGTCAT
ACCGCTGTGCTCTCCTCTCACATCGCGACGGGGCTAAAGTGCATCTCGGCACCCGCCCAACAGA
GAAACAGTACGAAACCCTGGAAAATCAGCTCGCGTTCCTGTGTCAGCAAGGCTTCTCCCTGGAG
AACGCACTGTACGCTCTGTCCGCCGTGGGCCACTTTACACTGGGCTGCGTATTGGAGGAACAGG
AGCATCAAGTAGCAAAAGAGGAAAGAGAGACACCTACCACCGATTCTATGCCCCCACTTCTGAAA
CAAGCAATTGAGCTGTTCGACCGGCAGGGAGCCGAACCTGCCTTCCTTTTCGGCCTGGAACTAA

TCATATGTGGCCTGGAGAAACAGCTAAAGTGCGAAAGCGGCGGGCCGACCGACGCCCTTGACG
ATTTTGACTTAGACATGCTCCCAGCCGATGCCCTTGACGACTTTGACCTTGATATGCTGCCTGCT
GACGCTCTTGACGATTTTGACCTTGACATGCTCCCCGGGTAAAGCGGCCGCGACTCTAGATCATA
ATCAGCCATACCACATTTGTAGAGGTTTTACTTGCTTTAAAAAACCTCCCACACCTCCCCCTGAAC
CTGAAACATAAAATGAATGCAATTGTTGTTGTTAACTTGTTTATTGCAGCTTATAATGGTTACAAAT
AAAGCAATAGCATCACAAATTTCACAAATAAAGCATTTTTTTCACTGCATTCTAGTTGTGGTTTGTC
CAAACTCATCAATGTATCTTAAGGGATCCCTAGAGGGACAGCCCCCCCCCAAAGCCCCCAGGGA
TGTAATTACGTCCCTCCCCCGCTAGGGGCAGCAGCGAGCCGCCCGGGGCTCCGCTCCGGTCCG
GCGCTCCCCCCGCATCCCCGAGCCGGCAGCGTGCGGGGACAGCCCGGGCACGGGGAAGGTGG
CACGGGATCGCTTTCCTCTGAACGCTTCTCGCTGCTCTTTGAGCCTGCAGACACCTGGGGGGAT
ACGGGGAAAAAGCTTTAGGCTGAAAGAGAGATTTAGAATGACAGAATCATAGAACGGCCTGGGTT
GCAAAGGAGCACAGTGCTCATCCAGATCCAACCCCCTGCTATGTGCAGGGTCATCAACCAGCAG
CCCAGGCTGCCCAGAGCCACATCCAGCCTGGCCTTGAATGCCTGCAGGGATGGGGCATCCACA
GCCTCCTTGGGCAACCTGTTCAGTGCGTCACCACCCTCTGGGGGAAAAACTGCCTCCTCATATC
CAACCCAAACCTCCCCTGTCTCAGTGTAAAGCCATTCCCCCTTGTCCTATCAAGGGGGAGTTTGC
TGTGACATTGTTGGTCTGGGGTGACACATGTTTGCCAATTCAGTGCATCACGGAGAGGCAGATCT
TGGGGATAAGGAAGTGCAGGACAGCATGGACGTGGGACATGCAGGTGTTGAGGGCTCTGGGAC
ACTCTCCAAGTCACAGCGTTCAGAACAGCCTTAAGGATAAGAAGATAGGATAGAAGGACAAAGAG
CAAGTTAAAACCCAGCATGGAGAGGAGCACAAAAAGGCCACAGACACTGCTGGTCCCTGTGTCT
GAGCCTGCATGTTTGATGGTGTCTGGATGCAAGCAGAAGGGGTGGAAGAGCTTGCCTGGAGAGA
TACAGCTGGGTCAGTAGGACTGGGACAGGCAGCTGGAGAATTGCCATGTAGATGTTCATACAAT
CGTCAAATCATGAAGGCTGGAAAAGCCCTCCAAGATCCCCAAGACCAACCCCAACCCACCCACC
GTGCCCACTGGCCATGTCCCTCAGTGCCACATCCCCACAGTTCTTCATCACCTCCAGGGACGGT
GACCCCCCCACCTCCGTGGGCAGCTGTGCCACTGCAGCACCGCTCTTTGGAGAAGGTAAATCTT
GCTAAATCCAGCCCGACCCTCCCCTGGCACAACGTAAGGCCATTATCTCTCATCCAACTCCAGGA
CGGAGTCAGTGAGGATGGGGCTGTCGACCTAGAGGGACAGCCCCCCCCCAAAGCCCCCAGGGA
TGTAATTACGTCCCTCCCCCGCTAGGGGCAGCAGCGAGCCGCCCGGGGCTCCGCTCCGGTCCG
GCGCTCCCCCCGCATCCCCGAGCCGGCAGCGTGCGGGGACAGCCCGGGCACGGGGAAGGTGG
CACGGGATCGCTTTCCTCTGAACGCTTCTCGCTGCTCTTTGAGCCTGCAGACACCTGGGGGGAT
ACGGGGAAAAAGCTTTAGGCTGAAAGAGAGATTTAGAATGACAGAATCATAGAACGGCCTGGGTT
GCAAAGGAGCACAGTGCTCATCCAGATCCAACCCCCTGCTATGTGCAGGGTCATCAACCAGCAG
CCCAGGCTGCCCAGAGCCACATCCAGCCTGGCCTTGAATGCCTGCAGGGATGGGGCATCCACA
GCCTCCTTGGGCAACCTGTTCAGTGCGTCACCACCCTCTGGGGGAAAAACTGCCTCCTCATATC
CAACCCAAACCTCCCCTGTCTCAGTGTAAAGCCATTCCCCCTTGTCCTATCAAGGGGGAGTTTGC
TGTGACATTGTTGGTCTGGGGTGACACATGTTTGCCAATTCAGTGCATCACGGAGAGGCAGATCT
TGGGGATAAGGAAGTGCAGGACAGCATGGACGTGGGACATGCAGGTGTTGAGGGCTCTGGGAC
ACTCTCCAAGTCACAGCGTTCAGAACAGCCTTAAGGATAAGAAGATAGGATAGAAGGACAAAGAG
CAAGTTAAAACCCAGCATGGAGAGGAGCACAAAAAGGCCACAGACACTGCTGGTCCCTGTGTCT
GAGCCTGCATGTTTGATGGTGTCTGGATGCAAGCAGAAGGGGTGGAAGAGCTTGCCTGGAGAGA
TACAGCTGGGTCAGTAGGACTGGGACAGGCAGCTGGAGAATTGCCATGTAGATGTTCATACAAT
CGTCAAATCATGAAGGCTGGAAAAGCCCTCCAAGATCCCCAAGACCAACCCCAACCCACCCACC
GTGCCCACTGGCCATGTCCCTCAGTGCCACATCCCCACAGTTCTTCATCACCTCCAGGGACGGT
GACCCCCCCACCTCCGTGGGCAGCTGTGCCACTGCAGCACCGCTCTTTGGAGAAGGTAAATCTT
GCTAAATCCAGCCCGACCCTCCCCTGGCACAACGTAAGGCCATTATCTCTCATCCAACTCCAGGA
CGGAGTCAGTGAGGATGGGGCTCAATTGTTTACTCCCTATCAGTGATAGAGAACGTATGAAGAGT
TTACTCCCTATCAGTGATAGAGAACGTATGCAGACTTTACTCCCTATCAGTGATAGAGAACGTATA
AGGAGTTTACTCCCTATCAGTGATAGAGAACGTATGACCAGTTTACTCCCTATCAGTGATAGAGAA
CGTATCTACAGTTTACTCCCTATCAGTGATAGAGAACGTATATCCAGTTTACTCCCTATCAGTGAT
AGAGAACGTATAAGCTTTAGGCGTGTACGGTGGGCGCCTATAAAAGCAGAGCTCGTTTAGTGAAC
CGTCAGATCGCCTGGAGCAATTCCACAACACTTTTGTCTTATACCAACTTTCCGTACCACTTCCTA
CCCTCGTAAAAAGCTTGTCCACCATGGCTCCTAAGAAAAAGCGGAAGGTGGACAAGAAATACTCA
ATCGGGCTGGACATCGGAACTAACTCAGTGGGGTGGGCAGTCATTACTGACGAGTACAAAGTGC
CAAGCAAGAAATTTAAGGTCCTGGGCAACACCGATAGGCACTCCATCAAGAAAAATCTGATTGGG
GCCCTGCTGTTCGACTCTGGAGAGACAGCTGAAGCAACTAGACTGAAAAGGACTGCTAGAAGGC

GCTATACCCGGCGAAAGAATCGCATCTGCTACCTGCAGGAGATTTTCTCTAACGAAATGGCCAAG
GTGGACGATAGTTTCTTTCATCGGCTGGAGGAATCATTCCTGGTCGAGGAAGATAAGAAACACGA
GAGACATCCTATCTTTGGAAACATTGTGGACGAGGTCGCTTATCACGAAAAATACCCCACCATCT
ATCATCTGCGCAAGAAACTGGTGGACTCTACAGATAAAGCAGACCTGCGGCTGATCTATCTGGCC
CTGGCTCACATGATTAAGTTCAGAGGCCATTTTCTGATCGAGGGAGATCTGAACCCAGACAATAG
CGATGTGGACAAGCTGTTCATCCAGCTGGTCCAGACATACAATCAGCTGTTTGAGGAAAACCCTA
TTAATGCATCTGGCGTGGACGCAAAAGCCATCCTGAGTGCCAGGCTGTCTAAGAGTAGAAGGCT
GGAGAACCTGATCGCTCAGCTGCCAGGCGAAAAGAAAAACGGCCTGTTTGGAAATCTGATTGCA
CTGTCACTGGGACTGACACCTAACTTCAAGAGCAATTTTGATCTGGCCGAGGACGCTAAACTGCA
GCTGAGCAAGGACACTTATGACGATGACCTGGATAACCTGCTGGCTCAGATCGGAGATCAGTAC
GCAGACCTGTTCCTGGCCGCTAAGAATCTGTCTGACGCTATCCTGCTGAGTGATATTCTGCGGGT
GAACACCGAGATTACAAAAGCCCCTCTGTCAGCTAGCATGATCAAGAGATATGACGAGCACCATC
AGGATCTGACCCTGCTGAAGGCACTGGTGCGCCAGCAGCTGCCCGAGAAGTACAAGGAAATCTT
CTTTGATCAGAGTAAGAACGGGTACGCCGGTTATATTGACGGCGGAGCTTCACAGGAGGAATTCT
ACAAGTTTATCAAACCTATTCTGGAGAAGATGGACGGCACCGAGGAACTGCTGGTGAAACTGAAT
CGCGAGGACCTGCTGCGCAAGCAGCGGACATTTGATAACGGCTCCATCCCCCACCAGATTCATC
TGGGAGAGCTGCACGCAATCCTGCGACGACAGGAAGACTTCTACCCATTTCTGAAGGATAACCG
CGAGAAGATCGAAAAAATTCTGACCTTCCGGATCCCTTACTATGTGGGGCCCCTGGCAAGGGGT
AATTCCCGCTTTGCCTGGATGACACGGAAATCTGAGGAAACAATCACTCCTTGGAACTTCGAGGA
AGTGGTCGATAAGGGAGCTTCCGCACAGTCTTTCATCGAGAGAATGACAAACTTCGACAAAAACC
TGCCAAATGAGAAAGTGCTGCCTAAGCACAGTCTGCTGTACGAGTATTTCACAGTCTATAACGAA
CTGACTAAGGTGAAATACGTCACCGAGGGGATGAGGAAGCCCGCCTTCCTGAGCGGTGAACAGA
AGAAAGCTATCGTGGACCTGCTGTTTAAAACCAATCGCAAGGTGACAGTCAAGCAGCTGAAGGA
GGACTACTTCAAGAAAATTGAATGTTTCGATTCTGTGGAGATCAGTGGCGTCGAAGACAGATTTAA
CGCTTCTCTGGGAACCTACCACGATCTGCTGAAGATCATTAAGGATAAAGACTTCCTGGACAACG
AGGAAAATGAGGATATCCTGGAAGACATTGTGCTGACCCTGACACTGTTTGAGGATCGCGAAATG
ATCGAGGAACGGCTGAAAACTTATGCCCATCTGTTCGATGACAAGGTGATGAAACAGCTGAAGCG
AAGAAGGTACACCGGCTGGGGACGACTGAGCAGAAAGCTGATCAACGGCATTCGGGACAAACA
GAGTGGAAAGACTATCCTGGACTTTCTGAAATCAGATGGCTTCGCTAACAGAAATTTTATGCAGCT
GATTCACGATGACAGCCTGACCTTCAAAGAGGATATCCAGAAGGCACAGGTGTCCGGGCAGGGT
GACTCTCTGCACGAGCATATCGCAAACCTGGCCGGGTCCCCCGCCATCAAGAAAGGTATTCTGC
AGACCGTGAAGGTGGTCGATGAGCTGGTGAAAGTCATGGGCAGGCATAAGCCAGAAAACATCGT
GATTGAGATGGCCCGCGAAAATCAGACCACACAGAAAGGACAGAAGAACAGCCGCGAGCGGAT
GAAAAGGATCGAGGAAGGCATTAAGGAACTGGGATCCCAGATCCTGAAAGAGCACCCTGTGGAA
AACACTCAGCTGCAGAATGAGAAGCTGTATCTGTACTATCTGCAGAATGGGCGGGATATGTACGT
GGACCAGGAGCTGGATATTAACCGACTGTCTGATTACGACGTGGATCATATCGTCCCACAGTCAT
TCCTGAAAGATGACAGCATTGACAATAAGGTGCTGACCCGGAGTGACAAAAACCGAGGAAAGAG
TGATAATGTCCCTTCAGAGGAAGTGGTCAAGAAAATGAAGAACTACTGGAGACAGCTGCTGAATG
CCAAACTGATCACACAGCGAAAGTTTGATAACCTGACTAAAGCTGAGAGAGGGGGTCTGTCAGAA
CTGGACAAAGCAGGCTTCATCAAGCGACAGCTGGTGGAGACCAGACAGATCACAAAGCACGTCG
CTCAGATTCTGGATAGCAGGATGAACACAAAGTACGATGAGAATGACAAACTGATCCGCGAAGTG
AAGGTCATTACTCTGAAGTCAAAACTTGTGAGCGACTTCAGAAAGGATTTCCAGTTCTACAAAGTC
AGGGAGATCAACAATTATCACCATGCTCATGACGCATACCTGAACGCAGTGGTCGGGACCGCCC
TGATTAAGAAATACCCCAAACTGGAGAGCGAATTCGTGTACGGTGACTATAAGGTGTACGATGTC
AGAAAAATGATCGCCAAGAGTGAGCAGGAAATTGGAAAAGCCACCGCTAAGTATTTCTTTTACTC
AAACATCATGAATTTCTTTAAGACTGAGATCACCCTGGCAAATGGGGAAATCCGAAAGAGACCAC
TGATTGAGACTAACGGCGAGACCGGAGAAATCGTGTGGGACAAGGGTAGGGATTTTGCCACAGT
GCGCAAGGTCCTGTCCATGCCTCAAGTGAATATTGTCAAGAAAACAGAGGTGCAGACTGGCGGA
TTCAGTAAGGAATCAATTCTGCCCAAACGGAACTCTGATAAGCTGATCGCCCGAAAGAAAGACTG
GGATCCCAAGAAATATGGGGGTTTCGACTCCCCAACAGTGGCTTACTCTGTCCTGGTGGTCGCA
AAGGTGGAGAAGGGGAAAAGCAAGAAACTGAAATCCGTCAAGGAGCTGCTGGGTATCACTATTA
TGGAGAGGAGCTCCTTCGAGAAGAACCCCATCGATTTTCTGGAGGCTAAAGGCTATAAGGAAGT
GAAGAAAGACCTGATCATTAAACTGCCAAAGTACAGCCTGTTTGAGCTGGAAAACGGAAGGAAGC
GAATGCTGGCATCCGCAGGAGAGCTGCAGAAGGGTAATGAACTGGCCCTGCCTTCTAAGTACGT

GAACTTCCTGTATCTGGCTAGCCACTACGAGAAGCTGAAAGGCTCCCCCGAGGATAACGAACAG
AAACAGCTGTTTGTGGAGCAGCACAAGCATTATCTGGACGAGATCATTGAACAGATTAGCGAGTT
CTCCAAAAGAGTGATCCTGGCTGACGCAAATCTGGATAAGGTCCTGAGCGCATACAACAAACACA
GAGATAAGCCAATCAGGGAGCAGGCCGAAAATATCATTCATCTGTTCACTCTGACCAACCTGGGA
GCCCCTGCAGCCTTCAAGTATTTTGACACTACCATCGATCGGAAACGATACACATCCACTAAGGA
GGTGCTGGACGCTACCCTGATTCACCAGAGCATTACCGGCCTGTATGAAACAAGGATTGACCTGT
CTCAGCTGGGGGGCGACCTCGAGGGAAGCGGAGAGGGCAGAGGAAGTCTGCTAACATGCGGT
GACGTCGAGGAGAATCCTGGCCCAGCACCGGGATCCATGGTGAGCAAGGGCGAGGAGCTGTTC
ACCGGGGTGGTGCCCATCCTGGTCGAGCTGGACGGCGACGTAAACGGCCACAAGTTCAGCGTG
TCCGGCGAGGGCGAGGGCGATGCCACCTACGGCAAGCTGACCCTGAAGTTCATCTGCACCACC
GGCAAGCTGCCCGTGCCCTGGCCCACCCTCGTGACCACCTTCACCTACGGCGTGCAGTGCTTC
GCCCGCTACCCCGACCACATGAAGCAGCACGACTTCTTCAAGTCCGCCATGCCCGAAGGCTACG
TCCAGGAGCGCACCATCTTCTTCAAGGACGACGGCAACTACAAGACCCGCGCCGAGGTGAAGTT
CGAGGGCGACACCCTGGTGAACCGCATCGAGCTGAAGGGCATCGACTTCAAGGAGGACGGCAA
CATCCTGGGGCACAAGCTGGAGTACAACTACAACAGCCACAAGGTCTATATCACCGCCGACAAG
CAGAAGAACGGCATCAAGGTGAACTTCAAGACCCGCCACAACATCGAGGACGGCAGCGTGCAG
CTCGCCGACCACTACCAGCAGAACACCCCCATCGGCGACGGCCCCGTGCTGCTGCCCGACAAC
CACTACCTGAGCACCCAGTCCGCCCTGAGCAAAGACCCCAACGAGAAGCGCGATCACATGGTCC
TGCTGGAGTTCGTGACCGCCGCCGGGATCACTCTCGGCATGGACGAGCTGTACAAGTAAACCTA
ATCTAGCAGCTCGCTGATCAGCCTCGACTGTGCCTTCTAGTTGCCAGCCATCTGTTGTTTGCCCC
TCCCCCGTGCCTTCCTTGACCCTGGAAGGTGCCACTCCCACTGTCCTTTCCTAATAAAATGAGGA
AATTGCATCGCATTGTCTGAGTAGGTGTCATTCTATTCTGGGGGGTGGGGTGGGGCAGGACAGC
AAGGGGGAGGATTGGGAAGACAATAGCAGGCATGCTGGGGATGCGGTGGGCTCTATGGCTTCT
GAGGCGGAAAGAACCAGCTGGGGCTCGATCCTCTAGTTGGCGCGTCATGGTCCATATGAATATC
CTCCTTAGTTCCTATTCCGCTAGCCTAGAGGGACAGCCCCCCCCCAAAGCCCCCAGGGATGTAA
TTACGTCCCTCCCCCGCTAGGGGCAGCAGCGAGCCGCCCGGGGCTCCGCTCCGGTCCGGCGC
TCCCCCCGCATCCCCGAGCCGGCAGCGTGCGGGGACAGCCCGGGCACGGGGAAGGTGGCACG
GGATCGCTTTCCTCTGAACGCTTCTCGCTGCTCTTTGAGCCTGCAGACACCTGGGGGGATACGG
GGAAAAAGCTTTAGGCTGAAAGAGAGATTTAGAATGACAGAATCATAGAACGGCCTGGGTTGCAA
AGGAGCACAGTGCTCATCCAGATCCAACCCCCTGCTATGTGCAGGGTCATCAACCAGCAGCCCA
GGCTGCCCAGAGCCACATCCAGCCTGGCCTTGAATGCCTGCAGGGATGGGGCATCCACAGCCT
CCTTGGGCAACCTGTTCAGTGCGTCACCACCCTCTGGGGGAAAAACTGCCTCCTCATATCCAACC
CAAACCTCCCCTGTCTCAGTGTAAAGCCATTCCCCCTTGTCCTATCAAGGGGGAGTTTGCTGTGA
CATTGTTGGTCTGGGGTGACACATGTTTGCCAATTCAGTGCATCACGGAGAGGCAGATCTTGGG
GATAAGGAAGTGCAGGACAGCATGGACGTGGGACATGCAGGTGTTGAGGGCTCTGGGACACTC
TCCAAGTCACAGCGTTCAGAACAGCCTTAAGGATAAGAAGATAGGATAGAAGGACAAAGAGCAAG
TTAAAACCCAGCATGGAGAGGAGCACAAAAAGGCCACAGACACTGCTGGTCCCTGTGTCTGAGC
CTGCATGTTTGATGGTGTCTGGATGCAAGCAGAAGGGGTGGAAGAGCTTGCCTGGAGAGATACA
GCTGGGTCAGTAGGACTGGGACAGGCAGCTGGAGAATTGCCATGTAGATGTTCATACAATCGTC
AAATCATGAAGGCTGGAAAAGCCCTCCAAGATCCCCAAGACCAACCCCAACCCACCCACCGTGC
CCACTGGCCATGTCCCTCAGTGCCACATCCCCACAGTTCTTCATCACCTCCAGGGACGGTGACC
CCCCCACCTCCGTGGGCAGCTGTGCCACTGCAGCACCGCTCTTTGGAGAAGGTAAATCTTGCTA
AATCCAGCCCGACCCTCCCCTGGCACAACGTAAGGCCATTATCTCTCATCCAACTCCAGGACGG
AGTCAGTGAGGATGGGGCTGGATCCGAAGCAGCTCCAGCCTACACAATCGCTCAAGACGTGTAA
TGCTTTTATTATATATTAGTCACGATATCTATAACAAGAAAATATATATATAATAAGTTATCACGTAA
GTAGAACATGAAATAACAATATAATTATCGTATGAGTTAAATCTTAAAAGTCACGTAAAAGATAATC
ATGCGTCATTTTGACTCACGCGGTCGTTATAGTTCAAAATCAGTGACACTTACCGCATTGACAAGC
ACGCCTCACGGGAGCTCCAAGCGGCGACTGAGATGTCCTAAATGCACAGCGACGGATTCGCGCT
ATTTAGAAAGAGAGAGCAATATTTCAAGAATGCATGCGTCAATTTTACGCAGACTATCTTTCTAGG
GTTAAAAAAGATTTGCGCTTTACTCGACCTAAACTTTAAACACGTCATAGAATCTTCGTTTGACAAA
AACCACATTGTGGGGTACCGAGCTCTTAATTAAGGCGCGCCGGGGAGGTTCCCTTTAGTGAGGG
TTAATTGCGGGTCGCCCTATAGTGAGTCGTATTACAATTCACTGGCCGTCGTTTTACAACGTCGT
GACTGGGAAAACCCTGGCGTTACCCAACTTAATCGCCTTGCAGCACATCCCCCTTTCGCCAGCTG
GCGTAATAGCGAAGAGGCCCGCACCGATCGCCCTTCCCAACAGTTGCGCAGCCTGAATGGCGAA

Supplementary Information

TGGCAAATTGTAAGCGTTAATATTTTGTTAAAATTCGCGTTAAATTTTTGTTAAATCAGCTCATTTTT
TAACCAATAGGCCGAAATCGGCAAAATCCCTTATAAATCAAAAGAATAGACCGAGATAGGGTTGA
GTGTTGTTCCAGTTTGGAACAAGAGTCCACTATTAAAGAACGTGGACTCCAACGTCAAAGGGCGA
AAAACCGTCTATCAGGGCGATGGCCCACTACGTGAACCATCACCCTAATCAAGTTTTTTGGGGTC
GAGGTGCCGTAAAGCACTAAATCGGAACCCTAAAGGGAGCCCCCGATTTAGAGCTTGACGGGGA
AAGCCGGCGAACGTGGCGAGAAAGGAAGGGAAGAAAGCGAAAGGAGCGGGCGCTAGGGCGCT
GGCAAGTGTAGCGGTCACGCTGCGCGTAACCACCACACCCGCCGCGCTTAATGCGCCGCTACA
GGGCGCGTCAG

Supplementary Information

Supplementary Table 1

Supplementary Table 1 - CrRNA sequences for genomic targets in human and mice.

Guide RNA	Target	crRNA Sequence
Cr1-GFAP	Human GFAP	GGGTGCCAGGACCCAGACGG
Cr2-GFAP	Human GFAP	GGAGACCCGGGCCAGTGAGC
Cr1-MYLIP	Human MYLIP	AGGGCAGAAACTGCTCAT
Cr2-MYLIP	Human MYLIP	TGGAAAACTATGGCATAGAA
Cr1-PIGM	Human PIGM	AACTTGAAGGTGGCTCCAGC
Cr2-PIGM	Human PIGM	TGTCCGTATACCTCACGTGC
Cr3-PIGM	Human PIGM	TGGATACTGCCATAGGCAGG
Cr1-p53	Mouse Trp53	GTGTAATAGCTCCTGCATGG
Cr1-MCT1	Human MCT1	CGTATAGTCATGATTGTTGG
Cr2-MCT1	Human MCT1	ACAGACGTATAGTTGCTGTA
Cr1-CDK12	Human CDK12	GTCGGTCAGTCCCCCTTACA
Cr2-CDK12	Human CDK12	GACGACCGTCTCCTGCTATA
Cr-Alu1	Alu	CCTGTAATCCCAGCA
Cr-Alu2	Alu	CACTTTGGGAGGCCG

Supplementary Information

Supplementary Table 2

Supplementary Table 2 - Primers and probes used in this study.

Target	Species	Function	Forward Primer	Reverse Primer
GFAP	Human	Cel1	TCATCATGGTCCAACCAACC	GAAGCGAACCTTCTCGATGT
MYLIP	Human	Cel1	CTCTTGGTGTCCTCCAGCAT	TTGGGCTAAAGTCATCTTTACAA
PIGM	Human	Cel1	AGTCTGCAGTCGTTTCGGTT	TCTATGGTTTCGCGGTGCAT
MCT1	Human	Cel1	TGTGAGGGAGCAGTTTCCTT	GCCAGCCATAAACTAATGCTTC
AAVS1	Human	3'INT	TGGCGTTACTATGGGAACATACG	CCAGATAGCACTGGGGACTCTTT
AAVS1	Human	5'INT	GTTTTTCTGGACAACCCCAAAGT	GCAATATCACGGGTAGCCAA
AAVS1	Human	WT	GTTTTTCTGGACAACCCCAAAGT	CCAGATAGCACTGGGGACTCTTT
PCSK9	Human	NGS	CCTGCAGGCCCAGGCTGC	TCCGCCCGGTACCGTGGA
Trp53	Mouse	Cel1	TAACAGCAGTCTCTGGGAGAAG	CTAAGCCCAAGAGGAAACAGAG
Kras	Mouse	NGS	AGGCCTGCTGAAAATGACTGAGTA	AAGCACGGATGGCATCTTGGACC
Trp53	Mouse	NGS	TGTAGTGAGGTAGGGAGCGAC	GAACCAAAGAGCGTTGGGC
Stk11	Mouse	NGS	GACACCTTCATCCACCGCA	TGGCACCTACTTCTTGACGTT

Supplementary Information

Supplementary Table 3

Supplementary Table 3 - Reagents for immunocytochemistry, immunohistochemistry, Western blotting and BaseScope

Antibody	Vendor	Catalog no.	clone	RRID Portal #
Cas9	CST, MA, USA	7A9-3A3	monoclonal	AB_2750916
beta actin	CST, MA, USA	8457	monoclonal	AB_10950489
anti mouse IgG (H+L) DyLight 488	ThermoFisher Scientific, MA, USA	35502	polyclonal	AB_844397
Cas9	Diagenode, Belgium	C15310258-20	polyclonal	AB_2715516
alpha SMA	CST, MA, USA	19245	D4K9N	AB_2734735
CD31	Abcam, Cambridge, UK	ab28364	polyclonal	AB_726362
F4/80	CST, MA, USA	70076	D2S9R	AB_2799771
CD45R	BD Pharmingen	553084	RA3-6B2	AB_394614
CD4	Abcam, Cambridge, UK	ab183685	EPR19514	AB_2686917
CD8	CST, MA, USA	98941	D4W2Z	AB_2756376
prosurfactant prot C (pSPC)	Abcam, Cambridge, UK	ab90716	polyclonal	AB_10674024
gH2AX(phospho-Histone)	CST, MA, USA	2577	Ser139	AB_2118010
Tenascin C	Millipore, MA, USA	AB19011	polyclonal	AB_2203804
NKx2.1 (TFF1)	Abcam, Cambridge, UK	ab76013	EP1584Y	AB_1310784
MAC2	Cedarlane, Canada	CL8942AP	M3/38	AB_10060357
Ki67	Abcam, Cambridge, UK	ab15580	polyclonal	AB_443209
pERK 1/2 (phospho-p42/44)	CST, MA, USA	4376	(Thr202/Tyr204) (20G11)	AB_331772
pMEK (Phospho-MEK1/2)	CST, MA, USA	2338	(Ser221) (166F8)	AB_490903
CRISPR/Cas9	Diagenode, Belgium	C15310258	polyclonal	AB_2715516
GFP	Abcam, Cambridge, UK	ab290	polyclonal	AB_303395
GAPDH	Abcam, Cambridge, UK	9485	polyclonal	AB_307275
MCT1 antibody	internally manufactured AstraZencea			
CDK12	CST, MA, USA	11973	polyclonal	AB_2715688
LKB1 / Stk11	CST, MA, USA	3047	D60C5,	AB_2198327
Trp53	CST, MA, USA	2524	1C12,	AB_331743
GAPDH	CST MA, USA	2118	14C10,	AB_561053
Vinculin	Sigma-Aldrich. MO, USA	V9131	hVIN-1,	AB_477629
goat anti-rabbit 800CW	LI-COR Biosciences, Cambridge, UK	925-32211	polyclonal	AB_2651127
donkey anti-rabbit 680RD	LI-COR Biosciences, Cambridge, UK	925-68073	polyclonal	AB_2716687

Probe set	Probe name	catalog number	Target
Kras G12D	I-BA-Mm-Kras-Donor-G12D-1zz-st	720508	Mus musculus v-Ki-ras2 Kirsten rat sarcoma viral oncogene homolog (KRAS), donor KrasG12D, mRNA
Kras G12C	I-BA-Mm-Kras-Donor-G12C-1zz-st	720518	Mus musculus v-Ki-ras2 Kirsten rat sarcoma viral oncogene homolog (KRAS), donor KrasG12C, mRNA

CST- Cell Signaling Technology

Supplementary Information

Supplementary Table 4

Supplementary Table 4 - sgRNAs used *in vivo.*

Primer target	Species	Method	Sequence sgRNA
PCSK9	Human	LNP delivery	CAGGTTCCACGGGATGCTCT
Kras	Mouse	AAV	GCAGCGTTACCTCTATCGTA
Trp53	Mouse	AAV	GTGTAATAGCTCCTGCATGG
Stk11	Mouse	AAV	ACTCCGAGACCTTATGCCGC
