# Supplementary material for: Development of an ObLiGaRe Doxycycline Inducible Cas9 system for pre-clinical cancer drug discovery
Source: Nat Commun. 2020 Sep 29;11:4903. doi: 10.1038/s41467-020-18548-9 (PMC7525522; doi:10.1038/s41467-020-18548-9)
Supplement: Supplementary file 3 — Reporting Summary [file 41467_2020_18548_MOESM3_ESM.pdf]

nature research

Corresponding author(s): Anders Lundin*, Michelle J. Porritt*, Himjyot Jaiswal, Frank Seeliger, Camilla Johansson, Abdel Wahad Bidar, Lukas Badertscher, Sandra Wimberger, Emma J. Davies, Elizabeth Hardaker, Carla P. Martins, Emily James, Therese Admyre, Amir Taheri-Ghahfarokhi, Jenna Bradley, Anna Schantz, Babak Alaeimahabadi, Maryam Clausen, Xiufeng Xu, Lorenz M. Mayr, Roberto Nitsch, Mohammad Bohlooly-Y, Simon T. Barry, Marcello Maresca*

Last updated by author(s): Jul 13, 2020

# Reporting Summary

Nature Research wishes to improve the reproducibility of the work that we publish. This form provides structure for consistency and transparency in reporting. For further information on Nature Research policies, see our Editorial Policies and the Editorial Policy Checklist.

## Statistics

For all statistical analyses, confirm that the following items are present in the figure legend, table legend, main text, or Methods section.

| n/a | Confirmed | |
|---|---|---|
| ☐ | ☒ | The exact sample size (*n*) for each experimental group/condition, given as a discrete number and unit of measurement |
| ☐ | ☒ | A statement on whether measurements were taken from distinct samples or whether the same sample was measured repeatedly |
| ☐ | ☒ | The statistical test(s) used AND whether they are one- or two-sided<br>*Only common tests should be described solely by name; describe more complex techniques in the Methods section.* |
| ☐ | ☒ | A description of all covariates tested |
| ☒ | ☐ | A description of any assumptions or corrections, such as tests of normality and adjustment for multiple comparisons |
| ☐ | ☒ | A full description of the statistical parameters including central tendency (e.g. means) or other basic estimates (e.g. regression coefficient) AND variation (e.g. standard deviation) or associated estimates of uncertainty (e.g. confidence intervals) |
| ☐ | ☒ | For null hypothesis testing, the test statistic (e.g. *F*, *t*, *r*) with confidence intervals, effect sizes, degrees of freedom and *P* value noted<br>*Give P values as exact values whenever suitable.* |
| ☒ | ☐ | For Bayesian analysis, information on the choice of priors and Markov chain Monte Carlo settings |
| ☒ | ☐ | For hierarchical and complex designs, identification of the appropriate level for tests and full reporting of outcomes |
| ☒ | ☐ | Estimates of effect sizes (e.g. Cohen's *d*, Pearson's *r*), indicating how they were calculated |

*Our web collection on statistics for biologists contains articles on many of the points above.*

## Software and code

Policy information about availability of computer code

| | |
|---|---|
| Data collection | ImageXpress Micro XLS Widefield Microscope (Molecular Devices, Sunnyvale, CA) or Incucyte Zoom (Essenbioscience, Michigan, US), FACS instrument BD LSR Fortessa (BD Bioscience), an iQue Screener PLUS (Intellicyt), confocal microscopy instrument Cell Voyager 7000S (CV7000S, Yokogawa, Japan), Illumina NextSeq500, Fragment Analyzer (Advanced Analytical Technologies), Discovery Ultra (Ventana Medical Systems, Inc, AZ, USA), NanoAssemblr (Precision NanoSystems Inc. Vancouver, Canada), Odyssey infrared imaging system and software (LI-COR Biosciences), Biospec 9.4T/20 MRI scanner (Bruker BioSpin, Karlsruhe, Germany) equipped with a 400 mT/m actively shielded gradient system with ParaVision (PV5.1) software, Qubit Fluorometer (Invitrogen) |
| Data analysis | Microsoft Excel, GraphPad Prism (v7.02), https://github.com/Ghahfarokhi/RIMA, ImageJ, Analyze 12.0 (Biomedical Imaging Resource, Mayo Clinic, Rochester, MN), FlowJo software (Becton, Dickinson and Company; 2019), featurecounts (version 1.4.4) and sailfish (version 0.9.0) within bcbio. Analyses was also performed using R (version 3.4.0, https://www.r-project.org/) and DESeq2 (version 1.14.1). https://github.com/lucapinello/CRISPResso |

For manuscripts utilizing custom algorithms or software that are central to the research but not yet described in published literature, software must be made available to editors and reviewers. We strongly encourage code deposition in a community repository (e.g. GitHub). See the Nature Research guidelines for submitting code & software for further information.

## Data

Policy information about availability of data

All manuscripts must include a data availability statement. This statement should provide the following information, where applicable:

- Accession codes, unique identifiers, or web links for publicly available datasets
- A list of figures that have associated raw data
- A description of any restrictions on data availability

All data that support the findings of this study are available from the corresponding authors upon reasonable request.

# Field-specific reporting

Please select the one below that is the best fit for your research. If you are not sure, read the appropriate sections before making your selection.

[x] Life sciences [ ] Behavioural & social sciences [ ] Ecological, evolutionary & environmental sciences

For a reference copy of the document with all sections, see nature.com/documents/nr-reporting-summary-flat.pdf

# Life sciences study design

All studies must disclose on these points even when the disclosure is negative.

| | |
|---|---|
| Sample size | For the in vitro work, Sample size was determined as generation of triple independent samples for comparisons between groups that is sufficient to perform statistical tests. The in vivo work, especially the teatment studies, sample size calculations were performed based on each models baseline tumor burden variability. |
| Data exclusions | No data was excluded from the studies reported in this manuscript |
| Replication | The experimental findings can be reliably reproduced. Some experiments providing key data were repeated by different co-authors. |
| Randomization | Animals were randomized to groups based on their weights measured prior to start of treatment in each of the experiments. |
| Blinding | The investigators were blinded to group allocation during data collection and analysis. The exception was with the nuclease assays - due to the need of visual organization of the groups to generate easily readable images. |

# Reporting for specific materials, systems and methods

We require information from authors about some types of materials, experimental systems and methods used in many studies. Here, indicate whether each material, system or method listed is relevant to your study. If you are not sure if a list item applies to your research, read the appropriate section before selecting a response.

### Materials & experimental systems

| n/a | Involved in the study | |
|---|---|---|
| [ ] | [x] | Antibodies |
| [ ] | [x] | Eukaryotic cell lines |
| [x] | [ ] | Palaeontology and archaeology |
| [ ] | [x] | Animals and other organisms |
| [x] | [ ] | Human research participants |
| [x] | [ ] | Clinical data |
| [x] | [ ] | Dual use research of concern |

### Methods

| n/a | Involved in the study | |
|---|---|---|
| [x] | [ ] | ChIP-seq |
| [ ] | [x] | Flow cytometry |
| [ ] | [x] | MRI-based neuroimaging |

## Antibodies

| | |
|---|---|
| Antibodies used | mouse Cas9 antibody (7A9-3A3 Cell Signaling Technologies) 1:800 dilution, goat anti-mouse DyLightTM 488 secondary antibody (ThermoFisher) 1:200, rabbit pAb CRISPR/Cas9 (C15310258, Diagenode, Liège, Belgium, 1:5000), rabbit pAb GFP (ab290; Abcam, Cambridge, UK, 1:5000), rabbit pAb GAPDH (9485; Abcam, Cambridge, UK, 1:10,000), rabbit mAb LKB1/Stk11 and mouse mAb p53 (3047 and 2524, Cell Signaling Technology, MA, USA; 1:5000), mAb vinculin (Sigma-Aldrich, MO, USA; 1:10,000), MCT1 antibody (internal AZ generated 1:1000), rabbit pAb CDK12 antibody (Cell Signaling Technology, MA, USA, #11973, 1:1000), rabbit mAb β-Actin (Cell Signaling Technology, MA, USA D6A8), rabbit mAb GAPDH (Cell Signaling Technology, 14C10, 1:3000), IRDye 800CW-labelled goat anti-rabbit (1:15,000) and 680RD-labelled donkey anti-rabbit (1:15,000) (LI-COR Biosciences, Cambridge, UK), Cas9 Diagenode, Liège, Belgium C15310258-20, alpha SMA Cell Signaling Technology, MA, USA 19245, CD31 Abcam, Cambridge, UK ab28364, F4/80 Cell Signaling Technology, MA, USA 70076, CD45R BD Pharmingen 553084, CD4 Abcam, Cambridge, UK ab183685, CD8 Cell Signaling Technology, MA, USA 98941, pSPC (prosurfactant prot C), Abcam, Cambridge, UK ab90716, gH2AX(phospho-Histone) Cell Signaling Technology, MA, USA 2577, Tenascin C Millipore, MA, USA AB19011, NKx2.1 (TFF1) Abcam, Cambridge, UK ab76013, MAC2 |

| | |
|---|---|
| | Cedarlane, Canada CL8942AP, Ki67 Abcam, Cambridge, UK, ab15580, pERK 1/2 (phospho-p42/44) Cell Signaling Technology, MA, USA 4376, pMEK (Phospho-MEK1/2) Cell Signaling Technology, MA, USA 2338, |
| Validation | MCT1 antibody (internal AZ generated and validated). All other antibodies used in this study are commercially available and were tested by the manufacturers to recognise the desired protein in a specific manner. |

# Eukaryotic cell lines

Policy information about cell lines

| | |
|---|---|
| Cell line source(s) | HEK293, HCT116, HepG2, OVACAR8, A549, M2-10B4 (M2), MC38 and Neuro2A were all purchased from ATCC (Manassas, VA, USA), this is indicated in the Methods section. ODInCas9 cell lines were generated by ZFN directed ObLiGaRe transgene integration, described in the Methods section |
| Authentication | Cell lines used have been authenticated 'in house' by AstraZeneca |
| Mycoplasma contamination | All cell lines have tested negative for mycoplasma |
| Commonly misidentified lines (See ICLAC register) | none used |

# Animals and other organisms

Policy information about studies involving animals; ARRIVE guidelines recommended for reporting animal research

| | |
|---|---|
| Laboratory animals | ODInCas9 mouse, on a C57BL/6N background, both males and females used in experiments, aged 8-18 weeks. AstraZeneca can share the animal model under a signed material transport agreement after that article is accepted for publication. |
| Wild animals | NA |
| Field-collected samples | NA |
| Ethics oversight | All mouse experiments were approved by the AstraZeneca internal committee for animal studies and the Gothenburg Ethics Committee for Experimental Animals (license numbers: 162–2015+ and 629-2017) compliant with EU directives on the protection of animals used for scientific purposes. This information is provided in the Methods section |

Note that full information on the approval of the study protocol must also be provided in the manuscript.

# Flow Cytometry

## Plots

Confirm that:

- [x] The axis labels state the marker and fluorochrome used (e.g. CD4-FITC).
- [x] The axis scales are clearly visible. Include numbers along axes only for bottom left plot of group (a 'group' is an analysis of identical markers).
- [x] All plots are contour plots with outliers or pseudocolor plots.
- [x] A numerical value for number of cells or percentage (with statistics) is provided.

## Methodology

| | |
|---|---|
| Sample preparation | Evaluation of cell population activation of ODInCas9 lines HEK293-C12.1 and N2A-C16 was performed 48 hr post Dox induction (0, 0.1 µg/ml, 1 µg/ml and 10 µg/ml). Cells were washed with PBS, detached in 2 mM EDTA (in 1x PBS) and resuspended in FACS buffer (2 mM EDTA, 2% FBS, 1x PBS). Cells were transferred in 96-well plates (Greiner #651201) and subjected to flow cytometry on an iQue Screener PLUS (Intellicyt). GFP positive cells were identified using a BL1 detector (530 nm) |
| Instrument | iQue Screener PLUS (Intellicyt) |
| Software | Data was analyzed with the ForeCyt software. |
| Cell population abundance | For the cytometric analysis, a minimum of 10000 events per replicate of each sample was recorded |
| Gating strategy | The threshold of the analyzer was adjusted in the corresponding channel of the flow cytometer to include live cell gating and exclude subcellular residues or cellular aggregates. GFP positive cells were identified using a BL1 detector (530 nm) |

- [x] Tick this box to confirm that a figure exemplifying the gating strategy is provided in the Supplementary Information.

# Magnetic resonance imaging

## Experimental design

Design type: Resting state acquisition

Design specifications: mice had maximum 5 imaging sessions, to measure tumor growth and establish baseline measurements and corresponding treatment measurements

Behavioral performance measures: NA

## Acquisition

Imaging type(s): structural

Field strength: Biospec 9.4T/20 MRI scanner (Bruker BioSpin, Karlsruhe, Germany) equipped with a 400 mT/m actively shielded gradient system

Sequence & imaging parameters: The imaging protocol consisted of three scans. First, an ungated gradient echo localizer scan (TR/TE/alpha: 175 ms/3.8 ms/20°, number of averages (NA): 2, field of view (FOV): 30x30 mm, matrix size: 128x128) to localize the lungs. Second, a set of two gated multi-slice coronal scans acquired with a fat suppressed Rapid Acquisition with Relaxation Enhancement (RARE) pulse sequence with the following parameters: TR/TE/RARE factor: 826 ms/9 ms/8, NA: 8, FOV: 30x30 mm, in-plane resolution: 117 x 117 µm, number of slices: 9-10, inter-slice: 1.4 mm and slice thickness: 0.7 mm. The total acquisition time was approximately 10 mins per scan. Slice position of the second RARE scan were interleaved into the interslice gap of the first RARE scan in order to cover the whole thoracic cavity without any slice gaps

Area of acquisition: The mouse was placed supine in a Plexiglas cradle and MRI acquisitions were synchronized with the respiratory cycle using a respiratory pad placed under the abdomen of the animal to minimize physiological artefacts (SA Instruments, Stony Brook, NY). Imaging was localized to the chest cavity

Diffusion MRI: ☐ Used ☒ Not used

## Preprocessing

Preprocessing software: Tumor nodules were segmented semi-automatically using the image analysis software Analyze 12.0 (Biomedical Imaging Resource, Mayo Clinic, Rochester, MN).

Normalization: NA

Normalization template: NA

Noise and artifact removal: MRI images are represented with inverted grayscale

Volume censoring: ImageJ (version 1.52a, NIH, MD, USA)

## Statistical modeling & inference

Model type and settings: NA

Effect(s) tested: NA

Specify type of analysis: ☐ Whole brain ☒ ROI-based ☐ Both

Anatomical location(s): Segmentation was performed as follows: 1) lung regions comprising tumor nodules were manually contoured in each slice, 2) tumor nodules in each defined region were then segmented based on histogram thresholding.

Statistic type for inference (See Eklund et al. 2016): Segmentation was performed as follows: 1) lung regions comprising tumor nodules were manually contoured in each slice, 2) tumor nodules in each defined region were then segmented based on histogram thresholding. Volumes of tumor nodules (in mm3) were calculated by multiplying the number of segmented voxels by the voxel volume resolution.

Correction: NA

## Models & analysis

| n/a | Involved in the study |
|---|---|
| ☒ | ☐ Functional and/or effective connectivity |
| ☒ | ☐ Graph analysis |
| ☒ | ☐ Multivariate modeling or predictive analysis |
